# Metabolism and toxicity of usnic acid and barbatic acid based on microsomes, S9 fraction, and 3T3 fibroblasts *in vitro* combined with a UPLC-Q-TOF-MS method

**DOI:** 10.3389/fphar.2023.1207928

**Published:** 2023-06-15

**Authors:** Hanxue Wang, Min Xuan, Juanjuan Diao, Nan Xu, Manlin Li, Cheng Huang, Changhong Wang

**Affiliations:** ^1^ Shanghai TCM-Integrated Hospital, Shanghai University of Traditional Chinese Medicine, Shanghai, China; ^2^ School of Pharmacy, Shanghai University of Traditional Chinese Medicine, Shanghai, China; ^3^ The MOE Key Laboratory for Standardization of Chinese Medicines, The SATCM Key Laboratory for New Resources and Quality Evaluation of Chinese Medicine, Shanghai Key Laboratory for TCM Complex Prescription, Institute of Chinese Materia Medica, Shanghai University of Traditional Chinese Medicine, Shanghai, China; ^4^ Department of Pharmacy, Qingdao Eighth People’s Hospital, Qingdao, China; ^5^ Analysis and Testing Center, Xinjiang Medical University (Xuelanshan Campus), Urumqi, China

**Keywords:** usnic acid, barbatic acid, metabolism, metabolic pathway, detoxification, UPLC/ESI-QTOF-MS

## Abstract

**Introduction:** Usnic acid (UA) and barbatic acid (BA), two typical dibenzofurans and depsides in lichen, have a wide range of pharmacological activities and hepatotoxicity concerns. This study aimed to clarify the metabolic pathway of UA and BA and illuminate the relationship between metabolism and toxicity.

**Methods:** An UPLC-Q-TOF-MS method was developed for metabolite identification of UA and BA in human liver microsomes (HLMs), rat liver microsomes (RLMs), and S9 fraction (RS9). The key metabolic enzymes responsible for UA and BA were identified by enzyme inhibitors combined with recombinant human cytochrome P450 (CYP450) enzymes. The cytotoxicity and metabolic toxicity mechanism of UA and BA were determined by the combination model of human primary hepatocytes and mouse 3T3 fibroblasts.

**Results:** The hydroxylation, methylation, and glucuronidation reactions were involved in the metabolic profiles of UA and BA in RLMs, HLMs, and RS9. CYP2C9, CYP3A4, CYP2C8, and UGT1A1 are key metabolic enzymes responsible for metabolites of UA and CYP2C8, CYP2C9, CYP2C19, CYP1A1, UGT1A1, UGT1A3, UGT1A7, UGT1A8, UGT1A9, and UGT1A10 for metabolites of BA. UA and BA did not display evident cytotoxicity in human primary hepatocytes at concentrations of 0.01–25 and 0.01–100 µM, respectively, but showed potential cytotoxicity to mouse 3T3 fibroblasts with 50% inhibitory concentration values of 7.40 and 60.2 µM.

**Discussion:** In conclusion, the attenuated cytotoxicity of BA is associated with metabolism, and UGTs may be the key metabolic detoxification enzymes. The cytotoxicity of UA may be associated with chronic toxicity. The present results provide important insights into the understanding of the biotransformation behavior and metabolic detoxification of UA and BA.

## Graphical Abstract

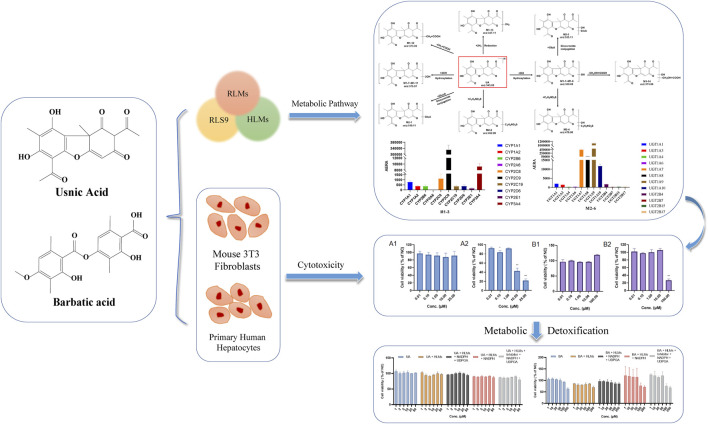

## 1 Introduction

Lichens, as one of the most fascinating organisms on Earth, are one of the longest-growing and most widely distributed plants; they grow throughout the northern temperate zones, especially the subarctic and coastal rainforests of Europe, Asia, and North America (Choudhary et al., 2005; Guo et al., 2008). As a valuable plant resource, the plants from genus *Usnea* have been widely used in fodder, dyes, food, perfumery, cosmetics, pharmaceuticals, preservatives, deodorants, ecological applications, and miscellaneous purposes throughout the world, particularly in Europe and East Asian countries, such as China, Japan, and India (Huang et al., 2009; Shukla et al., 2010; Bai et al., 2013; Srivastava et al., 2013; Prateeksha et al., 2016).

*Usnea*is is the filament plant of *Usnea diffracta* Vain and *Usnea longissima* Ach of the genus *Usnea*in Usneaceae family (Jiangsu New Medical College, 1977), and it has been well recorded in numerous monographs of medicine, such as the Shennong’s Herbal Classic of Materia Medica and Compendium of Materia Medica. *Usnea* has also been documented in the Drug Standard of the Ministry of Health of the People’s Republic of China, Uighur Medicine Fascicule, and in Chinese Materia Medica, Mongolian Medicine Volume, with diverse medicinal functions, such as clearing heat and detoxification, dispelling phlegm, relieving cough, regulating homeostasis and menstruation, and repelling insects (Chinese Pharmacopoeia Committee, 1999; Chinese Herbalism Editorial Board, 2004). With these versatile functions, *Usnea* is used to treat phlegm, malaria, cough, gasp, tuberculosis, headache, carbuncle, scrofula, acute mastitis, scalds, venomous snake bite, rheumatism, bruises, traumatic bleeding, and irregular menses. (Okuyama et al., 1995; Vijayakumar et al., 2000; Stickel et al., 2009; Honda et al., 2010; Ramos and Almeida, 2010; Backorova et al., 2012; Sokolov et al., 2012; Singh et al., 2013; Srivastava et al., 2013; Shtro et al., 2014; Avigan et al., 2016; Brown, 2017).

**FIGURE 1:**
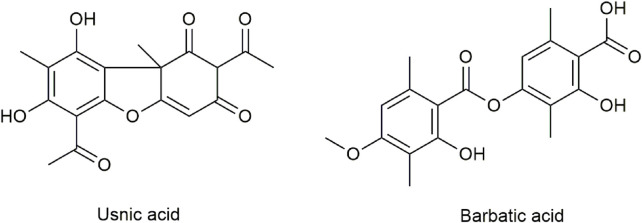
Chemical structures of UA and BA.

Modern pharmacological studies have confirmed that numerous secondary metabolites of *Usnea* have various biological activities and can be used as an antimicrobial (especially *Mycobacterium tuberculosis* and Gram-positive bacteria) (Honda et al., 2010; Ramos and Almeida, 2010), antipyretic–analgesic (Okuyama et al., 1995), anti-inflammatory (Vijayakumar et al., 2000), antitumor (Backorova et al., 2012; Singh et al., 2013) and antiviral medication (Sokolov et al., 2012; Shtro et al., 2014), for the promotion of wound healing (Burlando et al., 2009; Bruno et al., 2013; Zhang et al., 2018), photoprotection (Rancan et al., 2002; Kohlhardt-Floehr et al., 2010; Lohezic-Le et al., 2013; Kwong et al., 2020), antioxidative enzymes, and for the protection against mucosal damage (Vijayakumar et al., 2000; Halici et al., 2005; Bayir et al., 2006; Rabelo et al., 2012). All these health benefits of *Usnea* are due to the most common lichen compounds it contains, such as typical dibenzofuran compounds usnic acid (UA), longiusnine (−)-placodiolic acid, depsides barbatic acid (BA), evernic acid, diffractaic acid, and ramalic acid (Wang et al., 2022). Figure 1 shows the chemical structures of UA and BA.

However, recent reports associated with liver-related adverse events of UA-containing products aroused widespread concern (Wang et al., 2022), and the development of severe hepatotoxicity in a limited number of patients prompted the Food and Drug Administration to issue a warning letter, which led to the withdrawal of the product from the market in November 2001 (Frankos, 2005). In the past 2 decades, the hepatotoxicity caused by UA and its mechanism of action have been fully studied (Kwong and Wang, 2020; Wang et al., 2022). UA-induced hepatotoxicity is believed to have an idiosyncratic character, and the exact mechanism of toxicity has not been well defined. UA exerts cytotoxicity against rat hepatocytes by inducing the loss of membrane integrity and disruption of mitochondrial functions (Pramyothin et al., 2004). Oxidative stress induction has been indicated as a possible mode of toxicity of UA in mouse hepatocytes (Han et al., 2004), whereas in humans the toxicity of UA is observed in hepatocellular carcinoma cells (HepG2), the compound-induced DNA damage apoptosis, and cell cycle arrest (Chen et al., 2017). Most cellular studies imply that UA causes necrosis and affects mitochondrial functions (Araujo et al., 2015; Kwong et al., 2021).

Hepatic enzymes, especially cytochrome P450 (CYP450), commonly have an important role in the metabolism of drugs or chemicals. UA can significantly induce P450 activity in HepG2 cells at concentrations of 10 µM or higher, and slight UA metabolism by CYP1A1 may be present (Sahu et al., 2012). One study has demonstrated that CYP1A and CYP3A play an important role in reducing the rat hepatocellular toxicity of UA. CYP1A and CYP3A inhibitors alpha-naphthoflavone and ketoconazole can significantly enhance the hepatocellular toxicity of UA (Shi et al., 2014). An ultraperformance liquid chromatography-tandem mass spectrometry (UPLC-MS/MS) analysis used a trapping assay with GSH in human, rat, and mouse microsomes to identify four potential UA reactive metabolite formations, complexes derived from dehydrogenated and hydroxylated metabolites of UA and glutathione (GSH) The formation of UA reactive metabolites may be one of the mechanisms by which UA induces liver injuries. However, this suggestion was refuted by the conclusion that UA is detoxified by CYP1A and CYP3A (Piska et al., 2018).

Several studies studied UA metabolism *in vivo* and *in vitro*. However, no liver drug enzymes responsible for UA metabolism have been identified and confirmed, which resulted in an incomplete understanding of UA metabolites and metabolic pathways. In addition, there is an absence of direct evidence of the relationship between metabolism and attenuated toxicity of UA. For BA, another important depside component in *Usnea*, our previous pharmacokinetic results showed that the exposure of BA in rats was comparable to that of UA after oral administration of *Usnea* extract (Wang et al., 2018). The results indicated that BA is also a very important potential bioactive component in *Usnea*. However, studies on the metabolism and toxicity of BA, let alone the correlation between metabolism and toxicity, are limited. Therefore, metabolic pathway analysis of UA and BA must be conducted, and the correlation between metabolism and toxicity should be studied.

The *in vitro* metabolism toxicity prediction model can play an early warning role for toxicity. The combined model of co-cultures of human hepatocytes/liver microsomes and mouse 3T3 fibroblasts, with the characteristic of large flux, short cycle, and low cost, has evident advantages compared with the methods of synthesis, separation, and purification of metabolites and hepatocyte culture (Li et al., 2012).

This study aimed to use *in vitro* metabolic models of human liver microsomes (HLMs), rat liver microsomes (RLMs), and S9 fraction (RS9) combined with UPLC with quadrupole time-of-flight mass spectrometry (UPLC-Q-TOF-MS) technology to analyze and identify the metabolites and metabolic pathways of UA and BA. The method of enzyme inhibitor combined with recombinant human CYP450 enzymes was used to identify and confirm the metabolic enzymes responsible for UA and BA. Finally, the co-incubation model of HLMs and mouse 3T3 fibroblasts was used to evaluate the correlation between UA and BA metabolism and hepatocyte toxicity by the Cell Counting Kit-8 test and clarify the mechanism of toxicity induced or attenuated by metabolism. The results of this study will provide a basis for the systematic understanding of metabolic profiles and potential toxic mechanisms of UA and BA.

## 2 Materials and methods

### 2.1 Reagents and materials

UA (CAS 7562-61-0; batch number: DSTDS041601) with a purity of >98% was obtained from Chengdu Desite Biotechnology Co., Ltd. (Sichuan, China) and BA (CAS 125-46-2; batch number: WuXiNP03531) with a purity of >98% was obtained from Wuxi Apptec Co., Ltd. (Shanghai, China). D-Glucose 6-phosphate (G-6-P) disodium salt hydrate, glucose-6-phosphate dehydrogenase (G-6-P-DH) from leuconostoc mesenteroides, β-nicotinamide adenine dinucleotide phosphate disodium salt (NADP+), uridine 5′-diphospho-glucuronidation trisodium salt (UDPGA), 3′-phosphoadenosine-5′-phosphosulfate (PAPS), α-naphthoflavone, 8-methoxypsoralen, orphenadrine, sulfaphenazole, quinidine, 4-methylpyridine, neutral red, tamoxifen, and LC-MS-grade formic acid were purchased from Sigma-Aldrich (St. Louis, MO, United States). Furafylline, nootkatone, and ketoconazole were obtained from Shanghai Yuanye Bio-Technology Co., Ltd. (Shanghai, China). Quercetin and alamethicin were provided by J&K Scientific (Beijing, China). Montelukast was obtained from TCI Development Co., Ltd. (Shanghai, China). Tris-base was obtained from Shanghai Majorbio Bio-pharm Technology Co., Ltd. (Shanghai, China). Bradford reagent was purchased from Sangon Biotech Co., Ltd. (Shanghai, China). cDNA-expressed recombinant CYP1A1, CYP1A2, CYP2A6, CYP2B6, CYP2C8, CYP2C9, CYP2C19, CYP2D6, CYP2E1, and CYP3A4 Bactosomes were acquired from Cypex Limited (Scotland, United Kingdom). Dimethyl sulfoxide, MgCl2, and HCl were purchased from Sinopharm Chemical Reagent Co., Ltd. (Shanghai, China). Mouse 3T3 fibroblasts were provided by CobioerBiosciences Co., Ltd. (Nanjing, China). RLMs and their liver S9 fractions (RLS9) were prepared from pooled liver tissues by standard differential ultracentrifugation (Li et al., 2017). HLMs, primary human hepatocytes, InVitroGRO plating medium, and InVitroGRO incubation medium were provided by BioIVT& Elevating Science (New York, United States). Dulbecco’s Modified Eagle Medium was obtained from Gibco (Grand Island, United States). High-performance liquid chromatography (HPLC)-grade methanol and acetonitrile were secured from Fisher Scientific (New Jersey, United States). Ultra-pure water was filtered using the Milli-Q system (Millipore, Bedford, MA, United States). All other chemical reagents and solvents were of either analytical or HPLC grade. The experiments with animals were approved by the Ethics Committee on Animal Experimentation of the Shanghai University of Traditional Chinese Medicine (protocol PZSHUTCM211227013).

### 2.2 Instrument and analysis conditions

The metabolic profiling study was carried out using a Shimadzu 30A UPLC system (Shimadzu, Kyoto, Japan) coupled with AB SCIEX Triple TOF™ 5600+ system (AB Sciex, CA, United States) equipped with an electrospray ionization (ESI) source. Mass spectrometric detection was performed in negative mode. Nitrogen was used as the nebulizer and auxiliary gas. The flow rates of nebulizer gas (GS 1), heater gas (gas 2), and curtain gas were 55, 55, and 35 psi, respectively. The turbo spray temperature was 550°C. The ion spray voltage floating was −4500 V, and the declustering potential was −100 V. The collision energy (CE) was −35 eV, and the CE spread was set to 15 eV. The TOF scan range was set from m/z 100 to m/z 1200.

Chromatographic separations were conducted on an ACQUITY UPLC HSS T3 column (2.1 × 100 mm, 1.8 µm, Waters, United States). The mobile phase was a gradient system consisting of 0.1% formic acid in water (A) and acetonitrile (B) with a gradient elution system: 10% B (0–1.5 min), 10%–40% B (1.5–3 min), 40%–95% B (3–10 min), 95% B (10–11.5 min), 95%–5% B (11–11.6 min), and 5% B (11.6–12 min). The column temperature was set to 40°C, the flow rate to 0.3 mL/min, and the injection volume to 5 µL.

### 2.3 Incubation system

#### 2.3.1 Incubation systems of liver microsomes

For the phase I metabolism study, the incubation mixture, with a total volume of 200 μL, included liver microsomes (rat: 10.0 mg protein/mL or human: 21.9 mg protein/mL), 50 mM Buffer (Tris-HCl, pH 7.4), NADPH-generating system (10 mM G-6-P, 1 unit/mL G-6-P-DH, 4 mM MgCl2, and 1 mM NADP+), and substrate (1 µM). For the phase II metabolism study, the liver microsomes were first mixed with alamethicin (25 μg/mL), which was used as a perforating agent for 5 min preincubation at 37°C in a shaking water bath. The incubation system contained an additional 5 mM UDPGA, and the other reagents were the same as those in phase I. For the RLS9 metabolism study, as for the phase II metabolism study, the RLS9 was first mixed with alamethicin (25 μg/mL) for 5 min preincubation at 37°C in a shaking water bath. The incubation system contained additional 100 µM PAPS, and the other reagents were the same as those in phase I. The substrates were previously dissolved in methanol, and the final methanol concentration was below 1% (v/v) in the system. The reaction was initiated by the addition of a NADPH-generating system. After 60 min incubation at 37°C in a shaking water bath, the reaction was terminated by the addition of 1.0 mL ice-cold acetonitrile. The mixture was kept on ice until it was centrifuged at 12,000 × g for 10 min at 4°C. The supernatants were transferred and dried under a gentle stream of nitrogen at 37°C and then dissolved with 100 µL methanol for analysis. Blank control samples without substrates and negative control samples without NADP+ (for phase I metabolism study) or NADP+ and UDPGA (for phase II metabolism study) or NADP+ and PAPS (for RLS9 metabolism study) were included. Each group was prepared in triplicates.

#### 2.3.2 CYP phenotyping reaction

Chemical inhibitor methods together with recombinant CYP and UGT enzymes were used to identify the CYP and UGT isozymes involved in the metabolic pathways of UA and BA.

Chemical inhibition studies were performed by the addition of different human CYP inhibitors to the incubation mixture before the addition of the NADPH-generating system. The liver microsomes were first mixed with different concentrations of inhibitors and preincubated at 37°C for 5 min. The incubation system was similar to the phase I liver microsomal reaction system. The selective inhibitors of 10 major CYPs were as follows: α-naphthoflavone for CYP1A1, furafylline for CYP1A2, 8-methoxypsoralen for CYP2A6, orphenadrine for CYP2B6, quercetin for CYP2C8, sulfaphenazole for CYP2C9, quinidine for CYP2D6, nootkatone for CYP2C19, 4-methylpyridine for CYP2E1, and ketoconazole for CYP3A4. The concentrations of the 10 selective inhibitors were 5, 10, 20, 50, and 100 µM.

For the recombinant CYP and UGT enzyme method, the incubation system was similar to the phase I and II liver microsomal reaction systems. The liver microsomes or RLS9 were substituted with recombinant enzymes. The 10 recombinant CYP enzymes were as follows: CYP1A1, CYP1A2, CYP2A6, CYP2B6, CYP2C8, CYP2C9, CYP2D6, CYP2C19, CYP2E1, and CYP3A4. The 12 recombinant UGT enzymes were as follows: UGT1A1, UGT1A3, UGT1A4, UGT1A6, UGT1A7, UGT1A8, UGT1A9, UGT1A10, UGT2B4, UGT2B7, UGT2B15, and UGT2B17.

The subsequent steps were performed as described in Section 2.3.1.

### 2.4 Cytotoxicity assay

The cytotoxicity of UA and BA was assessed in primary human hepatocytes and mouse 3T3 fibroblasts seeded in 96-well plates, respectively. The concentrations of UA were 0.01, 0.1, 1, 10.0, and 25 μM, those of BA were 0.01, 0.1, 1, 10, and 100 μM, and tamoxifen (50 µM) was set as a positive control. The primary hepatocytes were incubated under 5% CO_2_ at an initial density of 7 × 10^5^ cells/mL per well at 37°C until the formation of a single-cell layer. The plating medium was changed to an incubation medium, and incubation was continued overnight. Mouse 3T3 fibroblasts were incubated under 5% CO_2_ at 37°C for 24 h. Different concentrations of UA or BA (100 µL) were incubated with human primary hepatocytes or mouse 3T3 fibroblasts for 48 h, respectively. At the end of incubation, the medium was removed quickly, and cells were washed once with 250 µL Hank’s balanced salt solution (HBSS). A total of 250 µL neutral red (250 μg/mL) was added for staining for 3 h. After neutral red was removed, 100 µL chromogenic solution was added, and shaking was carried out for 30 min at room temperature. The optical density (OD) of each well was read at 570 and 690 nm using a microplate reader, and the relative cell viability (% of normal control, NC) was calculated. Control samples without UA or BA and the positive control group were included. The amount of organic solvent was below 1% (v/v) in the system, and each group was prepared in triplicates.

### 2.5 Effect of metabolism on cytotoxicity

To determine the effect of metabolism on the cytotoxicity of UA and BA, fibroblast NIH mouse 3T3 fibroblasts were assessed and seeded in 96-well plates. The concentrations of UA were 1, 2, 5, 10, 25, and 50 μM, those of BA were 1, 10, 20, 50, 100, and 200 μM, and that of tamoxifen as positive control was 50 µM. Montelukast, sulfaphenazole, and ketoconazole were selected as exclusive inhibitors of CYP2C8, CYP2C9, and CYP3A4, respectively, and the concentrations were 100, 10, and 5 μM, respectively. Mouse 3T3 fibroblasts were incubated under 5% CO_2_ at 37°C overnight. At different concentrations of UA or BA, blank incubation medium (90 µL) as the control group and tamoxifen as the positive control group 1 were incubated with mouse 3T3 fibroblasts for 4 h. The HLMs were preincubated with different concentrations of UA or BA, NADPH, NADPH and UDPGA, or NADPH and UDPGA and inhibitors of CYP2C8, CYP2C9, and CYP3A4 at 37°C for 1 h. Positive control group 2 was also preincubated with HLMs together with NADPH and UDPGA. After preincubation, 90 µL supernatant was incubated with mouse 3T3 fibroblasts under 5% CO_2_ at 37°C for 4 h. The amount of organic solvent was below 1% (v/v) in the system, and each group was prepared in triplicates.

The subsequent steps were performed as described in Section 2.4.

### 2.6 Statistical analysis

All values were expressed as mean ± standard deviation (SD). The correlated fitting analyses were performed with Analyst TF 1.7.1, PeakView 2.2 (both belong to AB Sciex Pte. Ltd., MA, United States), and Graph pad Prism 6.0 (GraphPad Software Inc., CA, United States).

The cell viability was calculated as follows:

$$Cell\;viability\;\left(\%\right)=\frac{Treatment\;or\;positive\;control\;groups\;{OD}_{570}-{OD}_{690}}{Control\;group’s\;{OD}_{570}-{OD}_{690}}\times 100\%$$

The 50% inhibitory concentration (IC_50_) was calculated by Graph pad Prism as follows:

$$Y=Bottom+\frac{Top-Bottom}{1+10^{\left(\mathrm{Log}{IC}_{50}-X\right)\times HillSlope}}$$

Where Y and X are the relative activity and test concentrations, respectively.

## 3 Results

### 3.1 Identification and confirmation of UA biotransformation pathway *in vitro*

**FIGURE 2:**
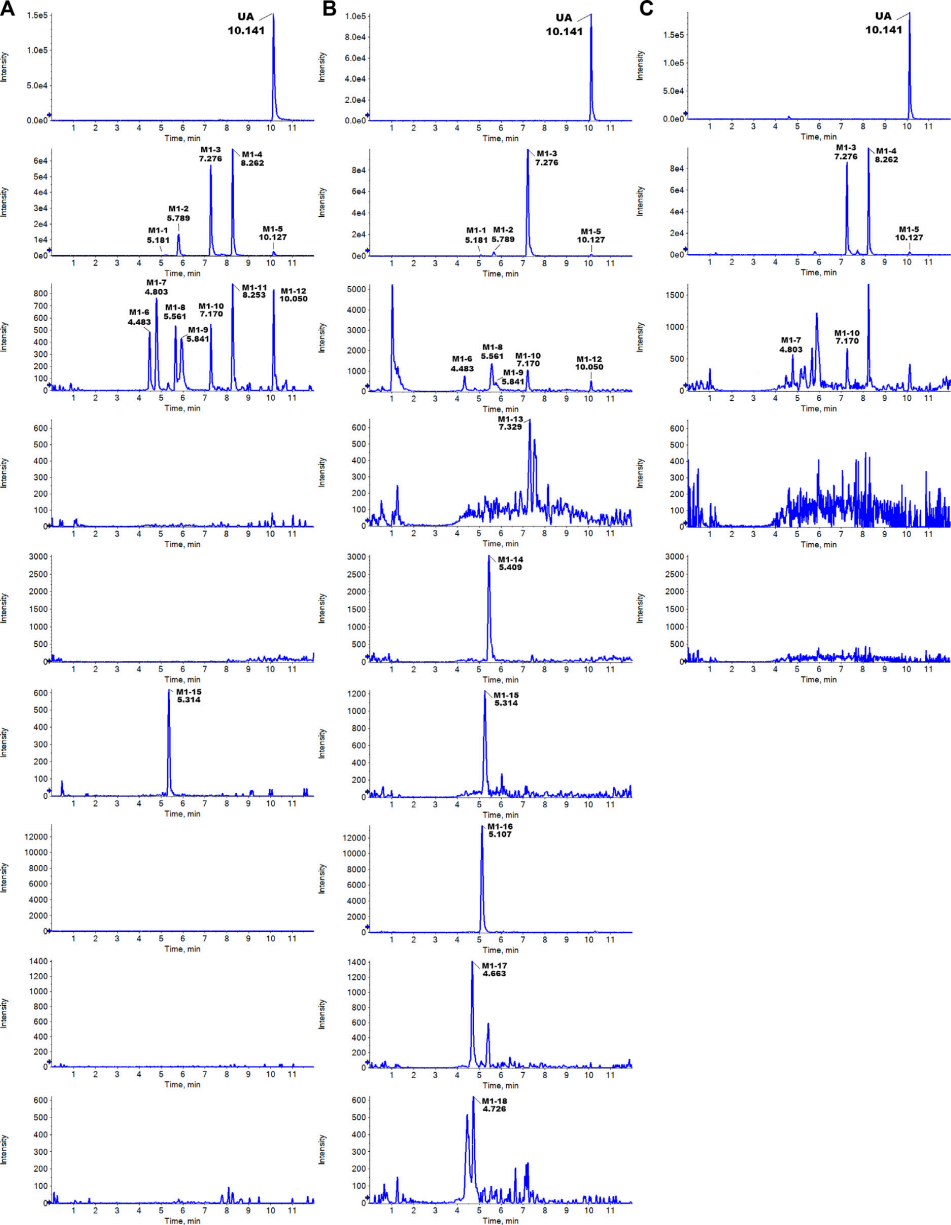
Typically extracted ion chromatograms of detected metabolites of UA from RLMs **(A)**, HLMs **(B)**, and RLS9 **(C)** *in vitro*.

**FIGURE 3:**
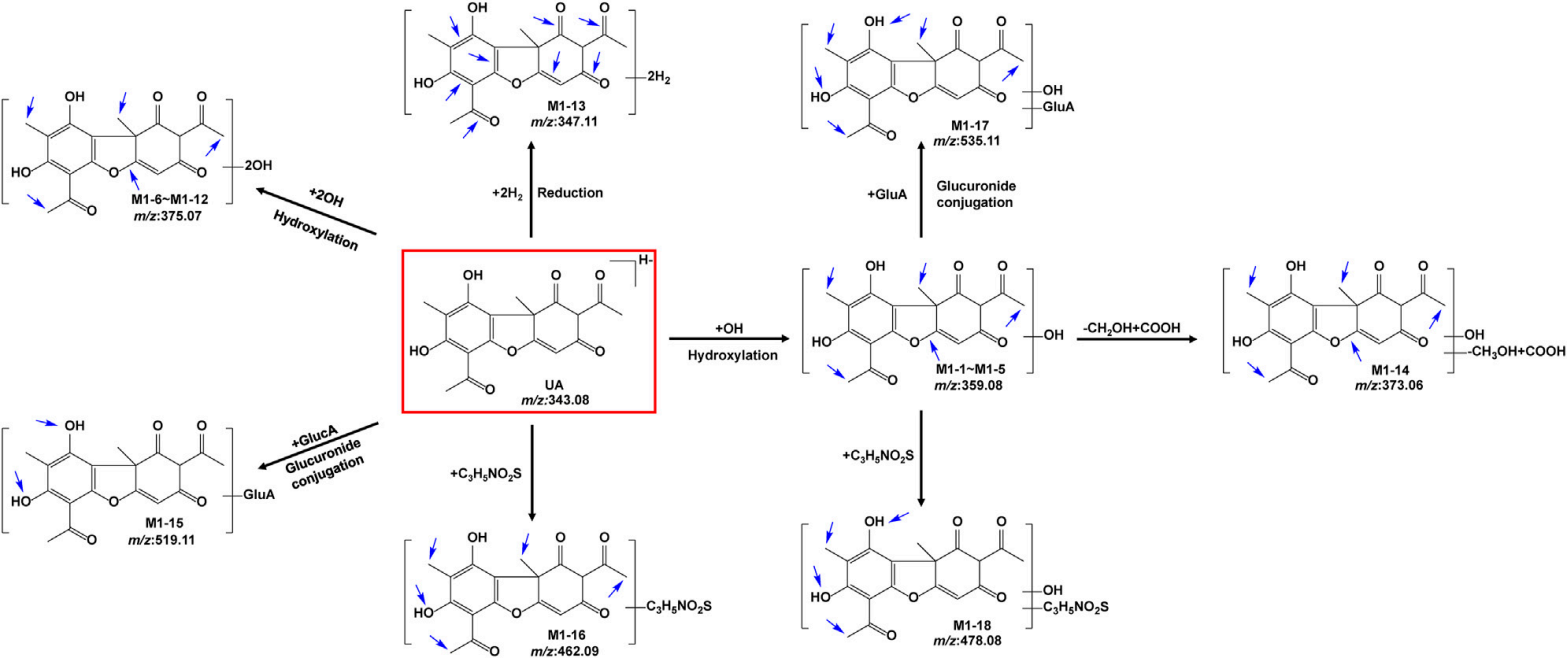
Proposed metabolic pathways of UA *in vitro* (positional isomerism has to be taken into account and the parent compound is marked by a rectangle. Blue arrows show the sites where metabolism is likely to occur).

The metabolites of UA were identified through a comparison of the blank control and drug-containing samples using UPLC-Q-TOF-MS technology. Most metabolites of UA were detected in the NADP + control group. Figure 2 provides the typically extracted ion chromatograms of all detected metabolites of UA from RLMs, HLMs, and RLS9. The biotransformation patterns of UA *in vitro* were speculated (Figure 3). Table 1 summarizes the retention time, measured and calculated mass, formula, and MS/MS fragmentation ions of all detected metabolites and UA.

**TABLE 1:** Metabolites information of UA in RLM, HLM, and RLS9.

Metabolites	Description	RT (min)	Formula	Calculated	Measured	Fragment ions	Source
			[M-H]-	mass	mass		
UA	parent	10.141	C_18_H_15_O_7_	343.0812	343.0851	328.0610, 313.0366, 299.0944, 259.0635, 231.0678, 215.0358, 189.0566, 83.0137	RLM, RLS9, HLM
M1-1	hydroxylation	5.181	C_18_H_15_O_8_	359.0772	359.0714	231.0711, 155.0363	RLM, HLM
M1-2	hydroxylation	5.789	C_18_H_15_O_8_	359.0772	359.0789	344.0534, 302.0453, 258.0190	RLM, HLM
M1-3	hydroxylation	7.276	C_18_H_15_O_8_	359.0772	359.0784	344.0562, 341.0686, 326.0448, 311.0207, 298.0495, 283.0273, 275.0577, 257.0481, 229.0490, 83.0146	RLM, RLS9, HLM
M1-4	hydroxylation	8.262	C_18_H_15_O_8_	359.0772	359.0825	344.0568, 329.0334, 315.0916, 298.0522, 285.0437, 275.0589, 257.0476, 247.0627, 229.0542, 217.0537, 213.0611, 201.0577, 189.0571, 83.0157	RLM, RLS9
M1-5	hydroxylation	10.127	C_18_H_15_O_8_	359.0772	359.0777	344.0504, 301.0324, 257.0518, 233.0431, 229.0509, 203.0630, 69.0318	RLM, RLS9, HLM
M1-6	2×hydroxylation	4.483	C_18_H_15_O_9_	375.0721	375.0753	360.0503, 342.0504, 300.0265, 155.0727, 143.0888, 87.0077, 59.0145	RLM, HLM
M1-7	2×hydroxylation	4.791	C_18_H_15_O_9_	375.0721	375.0714	360.0493, 318.0382, 259.0258, 143.0707	RLM, RLS9
M1-8	2×hydroxylation	5.561	C_18_H_15_O_9_	375.0721	375.0741	357.0603, 342.0385, 313.0380	RLM, HLM
M1-9	2×hydroxylation	5.841	C_18_H_15_O_9_	375.0721	375.0709	360.0564, 333.0579, 302.0489, 191.0847	RLM, HLM
M1-10	2×hydroxylation	7.170	C_18_H_15_O_9_	375.0721	375.0710	357.0656, 313.0715, 299.0150	RLM, RLS9, HLM
M1-11	2×hydroxylation	8.159	C_18_H_15_O_9_	375.0721	375.0780	359.0776, 331.0914, 313.0345, 291.0529, 247.0649, 217.0594	RLM
M1-12	2×hydroxylation	10.050	C_18_H_15_O_9_	375.0721	375.0707	235.0623, 233.0468, 207.0597, 141.0211	RLM, HLM
M1-13	di-reduction	7.329	C_18_H_19_O_7_	347.1136	347.1150	231.0616, 85.0290	HLM
M1-14	hydroxylation + oxidation	5.409	C_18_H_13_O_9_	373.0565	373.0594	355.0445, 340.0265, 327.1500, 271.0197, 243.0248, 185.0775, 87.0060	HLM
M1-15	glucuronidation	5.357	C_24_H_23_O_13_	519.1144	519.1149	343.0838, 328.0622, 259.0636	RLM, HLM
M1-16	cysteine conjugation	5.107	C_21_H_20_NO_9_S	462.0864	462.0858	341.0663, 326.0430, 257.0468	HLM
M1-17	hydroxylation + glucuronidation	4.663	C_24_H_23_O_14_	535.1088	535.1092	517.1005, 499.0336, 341.0666, 326.0424, 297.0799, 257.0451, 83.0111	HLM
M1-18	hydroxylation + cysteine conjugation	4.726	C_21_H_20_NO_10_S	478.0813	478.0836	432.2950, 341.0665, 326.0339	HLM

#### 3.1.1 Fragmentation studies of UA (m/z 343.0812)

**FIGURE 4:**
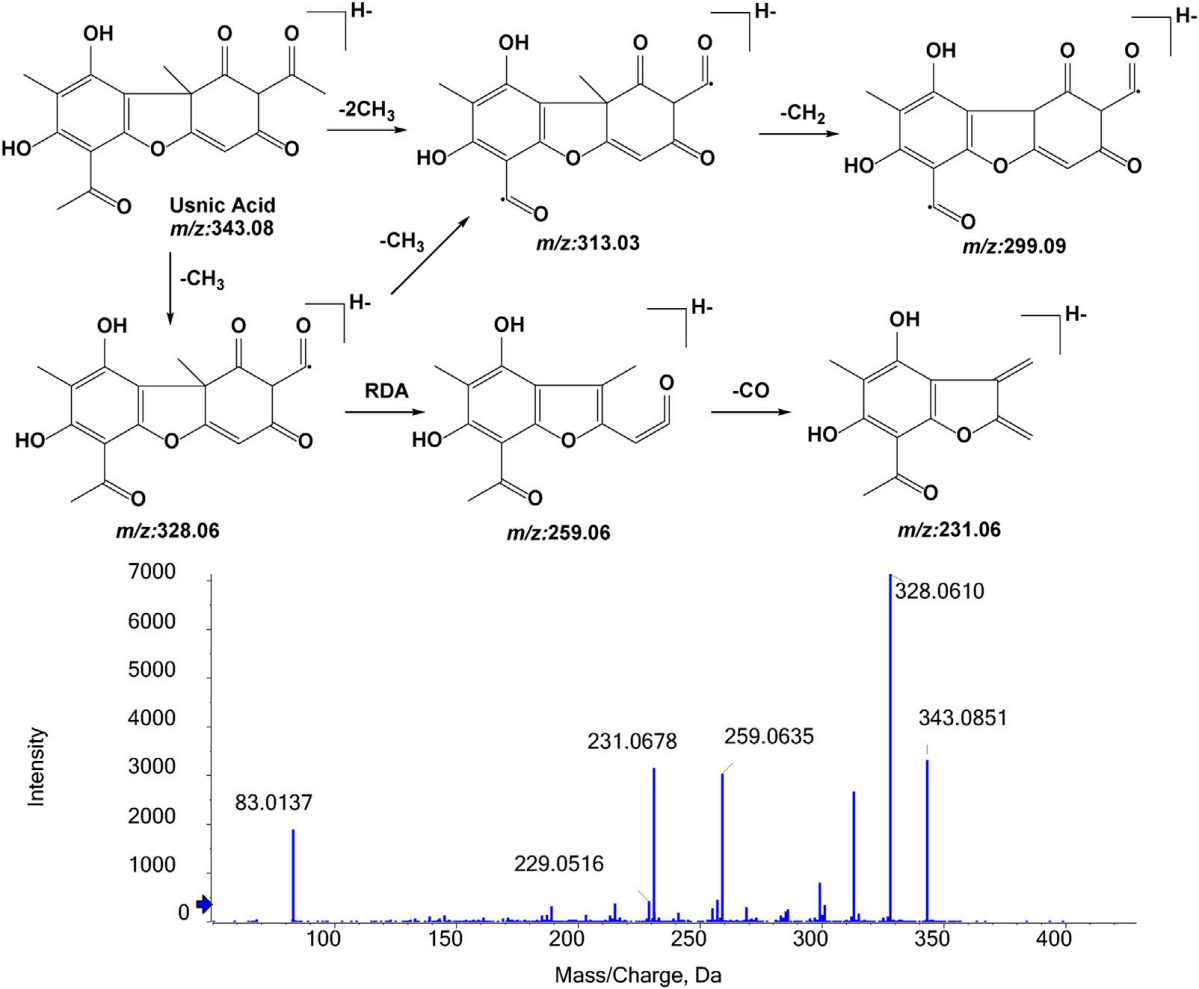
MS/MS spectrum of UA and its proposed fragmentation.

To identify its metabolites, we first investigated the MS/MS fragmentation behaviors of UA by UPLC/ESI-QTOF-MS. UA was eluted at 10.141 min and showed [M-H]^-^ at m/z 343.0851 (C_18_H_15_O_7_^−^, 11.3 ppm). UA provided abundant fragment ions at [M-H-CH_3_]^-^ m/z 328.0610 (C_17_H_12_O_7_^−^, 9.9 ppm) [M-H-2CH_3_]^-^ m/z 313.0366 (C_16_H_9_O_7_^−^, 7.4 ppm) [M-H-2CH_3_-CH_2_]^-^ m/z 299.0944 (C_15_H_7_O_7_^−^, 10.0 ppm) [M-H-CH_3_-C_3_HO_2_]^-^ m/z 259.0635 (C_14_H_11_O_5_^−^, 13.1 ppm), and [M-H-CH_3_-C_3_HO_2_-CO]^-^ m/z 231.0678 (C_13_H_11_O_4_^−^, 11.3 ppm). Figure 4 shows the proposed fragmentation pathways of UA.

#### 3.1.2 Metabolites of hydroxylation (m/z 359.0772)

Five hydroxylation metabolites of UA were identified in the microsomal incubation system *in vitro*. Metabolites **M1-1** (t_R_ = 5.181 min), **M1-2** (t_R_ = 5.789 min), **M1-3** (t_R_ = 7.276 min), **M1-4** (t_R_ = 8.262 min), and **M1-5** (t_R_ = 10.127 min) showed similar quasi-molecular of C_18_H_15_O_8_^−^ (m/z 359.0772 [M-H]^-^), which was 15.9949 Da higher than that of UA. In the MS/MS spectra, the [M-H]^-^ of **M1-1** showed fragment ions at [M-H-C_4_H_4_O_3_-CO]^-^ m/z 231.0711 (C_13_H_11_O_4_^−^) and [M-H-C_11_H_8_O_4_]^-^ m/z 155.0363 (C_7_H_7_O_4_^−^). The [M-H]^-^ of **M1-2** exhibited fragment ions at [M-H-CH_3_]^-^ m/z 344.0534 (C_17_H_12_O_8_^−^) [M-H-CH_3_-C_2_H_2_O]^-^ m/z 302.0453 (C_15_H_10_O_7_^−^), and [M-H-CH_3_-C_4_H_6_O_2_]^-^ m/z 258.0190 (C_13_H_6_O_6_^−^). The [M-H]^-^ of **M1-3** revealed fragment ions at [M-H-CH_3_]^-^ m/z 344.0562 (C_17_H_12_O_8_^−^) [M-H-OH-H]^-^ m/z 341.0686 (C_18_H_13_O_7_^−^) [M-H-CH_3_-OH-H]^-^ m/z 326.0448 (C_17_H_10_O_7_^−^) [M-H-CH_3_-OH-H-CO]^-^ m/z 298.0495 (C_16_H_10_O_6_^−^) [M-H-C_4_H_6_O_3_]^-^ m/z 257.0481 (C_14_H_9_O_5_^−^), and [M-H-C_4_H_6_O_3_-CO]^-^ 229.0490 (C_13_H_9_O_4_^−^). The [M-H]^-^ of **M1-4** presented fragment ions at [M-H-CH_3_]^-^ m/z 344.0568 (C_17_H_12_O_8_^−^) [M-H-2CH_3_]^-^ m/z 329.0334 (C_16_H_9_O_8_^−^) [M-H-CH_3_-OH-H-CO]^-^ m/z 298.0522 (C_16_H_10_O_6_^−^) [M-H-C_2_H_5_O_2_]^-^ m/z 285.0437 (C_16_H_10_O_6_^−^) [M-H-C_4_H_4_O_2_]^-^ m/z 275.0589 (C_14_H_11_O_6_^−^) [M-H-C_4_H_6_O_3_]^-^ m/z 257.0476 (C_14_H_9_O_5_^−^), and [M-H-C_4_H_6_O_3_-CO]^-^ m/z 229.0542 (C_13_H_9_O_4_^−^). The [M-H]^-^ of **M1-5** showed fragment ions at [M-H-CH_3_]^-^ m/z 344.0504 (C_17_H_12_O_8_^−^) [M-H-2CH_3_-CO]^-^ m/z 301.0324 (C_15_H_9_O_7_^−^) [M-H-CH_2_OH-CH_3_-2CO]^-^ m/z 257.0518 (C_14_H_9_O_5_^−^), and [M-H-C_4_H_6_O_3_-CO]^-^ m/z 229.0509 (C_13_H_9_O_4_^−^). The proposed fragmentation pathway of these metabolites, including the loss of methyl radical and retro Diels–Alder (RDA) transform, is similar to that of UA. In addition, the fragmentation behaviors between these metabolites were very similar. As no more information was observed, the specific structure of each compound could not be established from the mass spectrum data alone. Supplementary Figure S1 summarizes the MS/MS spectra and the proposed fragmentation pathway of the hydroxylation metabolites.

#### 3.1.3 Metabolites of dihydroxylation (m/z 375.0721)

Metabolites **M1-6** (t_R_ = 4.483 min), **M1-7** (t_R_ = 4.791 min), **M1-8** (t_R_ = 5.561 min), **M1-9** (t_R_ = 5.841 min), **M1-10** (t_R_ = 7.170 min), **M1-11** (t_R_ = 8.159 min), and **M1-12** (t_R_ = 10.050 min) showed similar quasi-molecular ion of C_18_H_15_O_9_^−^ (m/z 375.0721 [M-H]^-^), which was 31.9898 Da higher than that of UA. In the MS/MS spectra, the [M-H]^-^ of **M1-6** showed fragment ions at [M-H-CH_3_]^-^ m/z 360.0503 (C_17_H_12_O_9_^−^) and [M-H-CH_3_-C_2_H_4_O_2_]^-^ m/z 300.0265 (C_15_H_8_O_7_^−^). The [M-H]^-^ of **M1-7** exhibited fragment ions at [M-H-CH_3_]^-^ m/z 360.0493 (C_17_H_12_O_9_^−^) [M-H-CH_3_-CH_2_-CO]^-^ m/z 318.0382 (C_15_H_10_O_8_^−^), and [M-H-CH_3_-CH_2_-CO-C_2_H_2_O]^-^ m/z 259.0258 (C_13_H_7_O_6_^−^). The [M-H]^-^ of **M1-8** presented fragment ions at [M-H-OH-H]^-^ m/z 357.0603 (C_18_H_13_O_8_^−^) [M-H-OH-H-CH_3_]^-^ m/z 342.0385 (C_17_H_10_O_8_^−^), and [M-H-2CH_2_-2OH]^-^ m/z 313.0380 (C_16_H_9_O_7_^−^). The [M-H]^-^ of **M1-9** displayed fragment ions at [M-H-CH_3_]^-^ m/z 360.0564 (C_17_H_12_O_9_^−^) [M-H-CH_2_-CO]^-^ m/z 333.0579 (C_16_H_13_O_8_^−^), and [M-H-CH_3_-CH_2_-CO]^-^ m/z 302.0489 (C_15_H_10_O_7_^−^). The [M-H]^-^ of **M1-10** manifested fragment ions at [M-H-OH-H]^-^ m/z 357.0656 (C_18_H_13_O_8_^−^) [M-H-CO-2OH]^-^ m/z 313.0715 (C_17_H_13_O_6_^−^), and [M-H-2CH_3_-CO-OH-H]^-^ m/z 299.0150 (C_15_H_7_O_7_^−^). The [M-H]^-^ of **M1-11** showed fragment ions at [M-H-O]^-^ m/z 359.0776 (C_18_H_15_O_8_^−^) [M-H-CO-O]^-^ m/z 331.0914 (C_17_H_15_O_7_^−^) [M-H-C_2_H_6_O-O]^-^ m/z 313.0345 (C_16_H_9_O_7_^−^), and [M-H-CO-O-C_4_H_4_O_2_]^-^ m/z 247.0649 (C_13_H_11_O_5_^−^). The [M-H]^-^ of **M1-12** indicated fragment ions at [M-H-C_6_H_5_O_4_^3-^]^-^ m/z 235.0623 (C_12_H_11_O_5_^−^) [M-H-C_6_H_7_O_4_^3-^]^-^ m/z 233.0468 (C_12_H_9_O_5_^−^) [M-H-C_6_H_5_O_4_^3-^-CO]^-^ m/z 207.0597 (C_11_H_11_O_4_^−^), and [M-H-C_12_H_11_O_5_^3-^]^-^ m/z 141.0211 (C_6_H_5_O_4_^−^). **M1-6** to **M1-12** were tentatively identified as a metabolite of 2 × hydroxylation. Supplementary Figure S2 summarizes the MS/MS spectra and the proposed fragmentation pathway of the 2 × hydroxylation metabolites.

#### 3.1.4 Metabolites of di-reduction (m/z 347.1136)

Metabolite **M1-13** was detected at 7.329 min and showed quasi-molecular at m/z 347.1150 ([M-H]^-^, C_18_H_19_O_7_^−^, 7 ppm). In the MS/MS spectra, the [M-H]^-^ of **M1-13** showed fragment ions at [M-H-C_4_H_4_O_2_-CH_3_-OH]^-^ m/z 231.0616 (C_13_H_11_O_4_^−^, −15.5 ppm) and [M-H-C_14_H_14_O_5_]^-^ m/z 85.0290 (C_4_H_5_O_2_^−^, 7 ppm). The proposed fragmentation pathway of **M1-13** revealed similar characteristics to that of UA, including the RDA transform. Supplementary Figure S3 exhibits the MS/MS spectra and the proposed fragmentation pathway.

#### 3.1.5 Metabolites of hydroxylation and oxidation (m/z 373.0565)

Metabolite **M1-14** was detected at 5.409 min and showed quasi-molecular at m/z 373.0594 ([M-H]^-^, C_18_H_13_O_9_^−^, 10.7 ppm). In the MS/MS spectra, the [M-H]^-^ of **M1-14** exhibited fragment ions at [M-H-OH-H]^-^ m/z 355.0445 (C_18_H_11_O_8_^−^, −1.0 ppm) [M-H-CH_3_-CO-H]^-^ m/z 329.0334 (C_16_H_9_O_8_^−^, 12.8 ppm) [M-H-CO-OH-H]^-^ m/z 327.0495 (C_17_H_11_O_7_^−^, −1.3 ppm) [M-H-C_3_H_4_O-CO-OH-H]^-^ m/z 271.0197 (C_14_H_7_O_6_^−^, −14.8 ppm), and [M-H-C_3_H_4_O-2CO-OH-H]^-^ m/z 243.0248 (C_13_H_7_O_5_^−^, −16.5 ppm). **M1-14** was tentatively identified as a metabolite of hydroxylation and oxidation. Supplementary Figure S4 presents the MS/MS spectra and the proposed fragmentation pathway of **M1-14**.

#### 3.1.6 Metabolites of glucuronidation (m/z 519.1144)

Metabolite **M1-15** presented a quasi-molecular ion at m/z 519.1149 ([M-H]^-^, C_24_H_23_O_13_^−^, 7.3 ppm) and could be detected at 5.357 min, which was 176.0321 Da higher than that of UA m/z 343.0851 (C_18_H_15_O_7_^−^, 11.3 ppm) and indicates the characteristic loss of glucuronic acid group (C_6_H_10_O_7_-H_2_O). The MS/MS spectra exhibited fragment ions at [M-H-C_6_H_8_O_6_]^-^ m/z 343.0838 (C_18_H_15_O_7_^−^, 7.5 ppm) [M-H-C_6_H_8_O_6_-CH_3_]^-^ m/z 328.0622 (C_17_H_12_O_7_^−^, 13.6 ppm), and [M-H-C_6_H_8_O_6_-CH_3_-C_3_HO_2_]^-^ m/z 259.0636 (C_14_H_11_O_5_^−^, −13.5 ppm). The proposed fragmentation pathway of metabolites **M1-15** was similar to that of UA, including the loss of methyl radical and RDA transform. As no more information was observed, the specific structure could not be established from the mass spectrum data alone. Supplementary Figure S5 shows the MS/MS spectra and the proposed fragmentation pathway of **M1-15**.

#### 3.1.7 Metabolites of cysteine conjugation (m/z 462.0864)

Metabolite **M1-16** showed a quasi-molecular ion at m/z 462.0858 ([M-H]^-^, C_21_H_20_NO_9_S^−^, −4.2 ppm) and could be detected at 5.107 min. The MS/MS spectra displayed fragment ions at [M-H-CH_3_]^-^ m/z 447.0627 (C_20_H_17_NO_9_S^−^, 1.9 ppm) [M-H-C_3_H_5_NO_2_S-2H]^-^ m/z 341.0663 (C_18_H_13_O_7_^−^, 2.1 ppm) [M-H-CH_3_-C_3_H_5_NO_2_S-2H]^-^ m/z 326.0430 (C_17_H_10_O_7_^−^, 2.7 ppm), and [M-H-C_3_H_5_NO_2_S-2H-CH_3_-C_3_HO_2_]^-^ m/z 257.0468 (C_14_H_9_O_5_^−^, 9.1 ppm). The proposed fragmentation pathway of metabolites **M1-16** was similar to that of UA, including the loss of methyl radical and RDA transform. As no more information was observed, the specific structure could not be established from the mass spectra data alone. Supplementary Figure S6 contains the MS/MS spectra and the proposed fragmentation pathway of **M1-16**.

#### 3.1.8 Metabolites of hydroxylation and glucuronidation (m/z 535.1088)

Metabolite **M1-17** manifested the quasi-molecular ion at m/z 535.1092 ([M-H]^-^, C_24_H_23_O_14_^−^, 1.8 ppm) and could be detected at 4.663 min.The MS/MS spectra revealed fragment ions at [M-H-OH-H]^-^ m/z 517.1005 (C_24_H_21_O_13_^−^, 5.5 ppm) [M-H-OH-H-C_6_H_8_O_6_]^-^ m/z 341.0666 (C_18_H_13_O_7_^−^, 3.0 ppm) [M-H-OH-H-C_6_H_8_O_6_-CH_3_]^-^ m/z 326.0424 (C_17_H_10_O_7_^−^, 0.9 ppm) [M-H-2OH-C_6_H_8_O_6_-CO]^-^ m/z 297.0799 (C_17_H_13_O_5_^−^, 14.0 ppm), and [M-H-OH-H-C_6_H_8_O_6_-C_3_HO_2_-CH_3_]^−^m/z 257.0451 (C_14_H_9_O_5_^−^, 0.9 ppm). The proposed fragmentation pathway of metabolite **M1-17** was similar to that of UA, including the loss of methyl radical and RDA transform. As no more information was observed, the specific structure could not be established from the mass spectra data alone. Supplementary Figure S7 exhibits the MS/MS spectra and the proposed fragmentation pathway of **M1-17**.

#### 3.1.9 Metabolites of hydroxylation and cysteine conjugation (m/z 478.0813)

Metabolite **M1-18** unveiled a quasi-molecular ion at m/z 478.0836 ([M-H]^-^, C_21_H_20_NO_10_S^−^, 1.8 ppm) and could be detected at 4.726 min. The MS/MS spectra showed fragment ions at [M-H-C_3_H_5_NO_2_S-OH-H]^–^ m/z 341.0665 (C_18_H_13_O_7_^−^, 2.7 ppm), and [M-H-C_8_H_8_O_3_]^-^ m/z 326.0339 (C_13_H_12_NO_7_S^−^, 3.1 ppm). As no more information was observed, the specific structure could not be established from the mass spectra data alone. Supplementary Figure S8 displays the MS/MS spectra and the proposed fragmentation pathway of **M1-18**.

### 3.2 Identification and confirmation of BA biotransformation pathway *in vitro*

**FIGURE 5:**
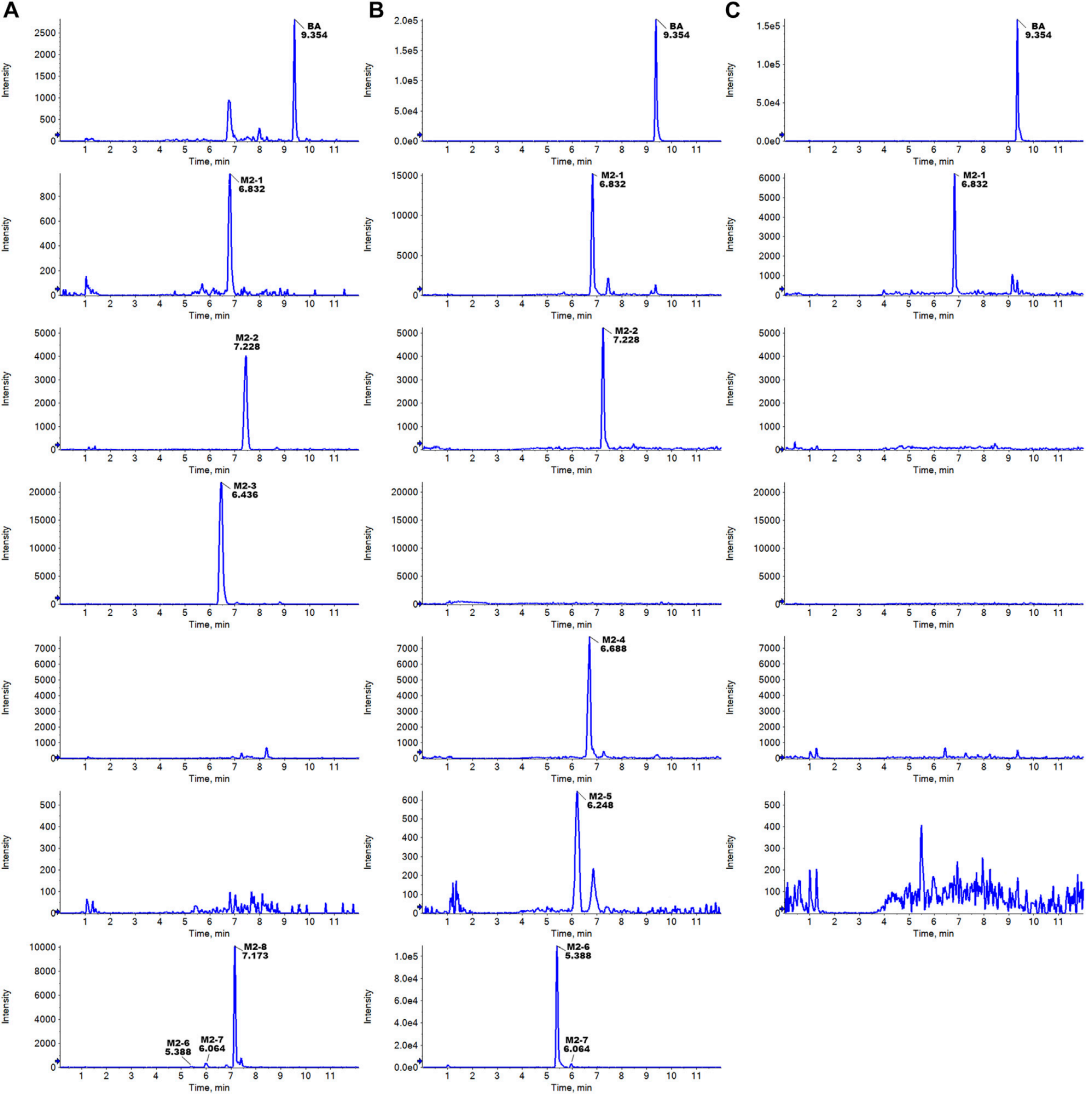
Typically extracted ion chromatograms of detected metabolites of BA from RLMs **(A)**, HLMs **(B)**, and RLS9 **(C)** *in vitro*.

**FIGURE 6:**
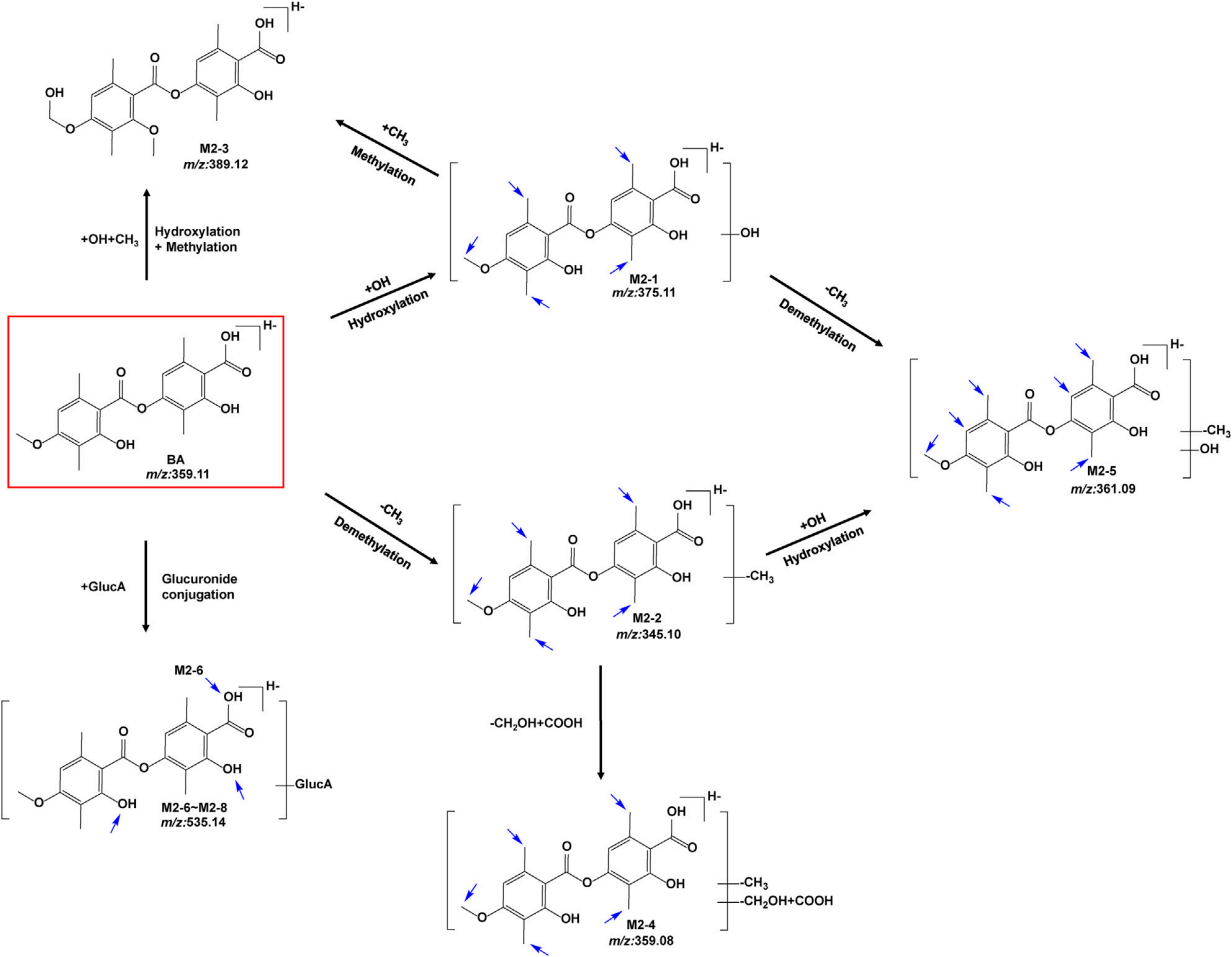
Proposed metabolic pathways of BA *in vitro* (positional isomerism has to be taken into account and the parent compound is marked by a rectangle. Blue arrows show the sites where metabolism is likely to occur).

The metabolites of BA were identified through a comparison of the blank control and drug-containing samples using UPLC-Q-TOF-MS technology. Figure 5 displays the typically extracted ion chromatograms of all detected metabolites of BA from RLMs, HLMs, and RLS9. The biotransformation patterns of BA *in vitro* were speculated (Figure 6). Table 2 summarizes the retention time, measured and calculated mass, formula, and MS/MS fragmentation ions of all detected metabolites and BA.

**TABLE 2:** Metabolites information of BA in RLM, HLM, and RLS9.

Metabolites	Description	RT (min)	Formula	Calculated	Measured	Fragment ions	Source
			[M-H]-	mass	mass		
BA	Parent	9.354	C_19_H_19_O_7_	359.1136	359.1103	181.0506, 163.0398, 137.0601, 119.0494	RLM, RLS9, HLM
M2-1	hydroxylation	6.832	C_19_H_19_O_8_	375.1074	375.1109	181.0514, 163.0411, 137.0614, 119.0503, 93.0728	RLM, RLS9, HLM
M2-2	demethylation	7.228	C_18_H_17_O_7_	345.0974	345.0989	311.2200, 181.0515, 163.0394, 137.0602, 119.0512, 87.0097	RLM, HLM
M2-3	hydroxylation + methylation	6.436	C_20_H_21_O_8_	389.1242	389.1260	345.1334, 313.1101, 298.0836, 283.0972, 268.0761, 225.0790, 181.0854, 151.0773, 121.0658	RLM
M2-4	demethylation + carbonylation	6.688	C_18_H_15_O_8_	359.0772	359.0786	341.0671, 313.0701, 297.0765, 285.0728, 270.0519, 259.0934, 241.0870, 228.0370, 213.0582, 177.0269, 163.0394, 137.0603, 121.0691, 81.0719, 59.0140, 57.0339	HLM
M2-5	hydroxylation + demethylation	6.248	C_18_H_17_O_8_	361.0929	361.0904	197.0484, 181.0495, 179.0344, 163.0393, 137.0604, 135.0454, 119.0492	HLM
M2-6	glucuronidation	5.388	C_25_H_27_O_13_	535.1452	535.1456	491.1557, 359.1139, 315.1228, 181.0511, 163.0404, 137.0610, 113.0241	RLM, HLM
M2-7	glucuronidation	6.064	C_25_H_27_O_13_	535.1457	535.1419	359.1136, 313.0950, 181.0512, 163.0405, 137.0608, 113.0252	RLM, HLM
M2-8	glucuronidation	7.173	C_25_H_27_O_13_	535.1457	535.1432	514.9668, 359.1099, 181.0495, 163.0419	RLM

#### 3.2.1 Fragmentation studies of BA (m/z 359.1136)

**FIGURE 7:**
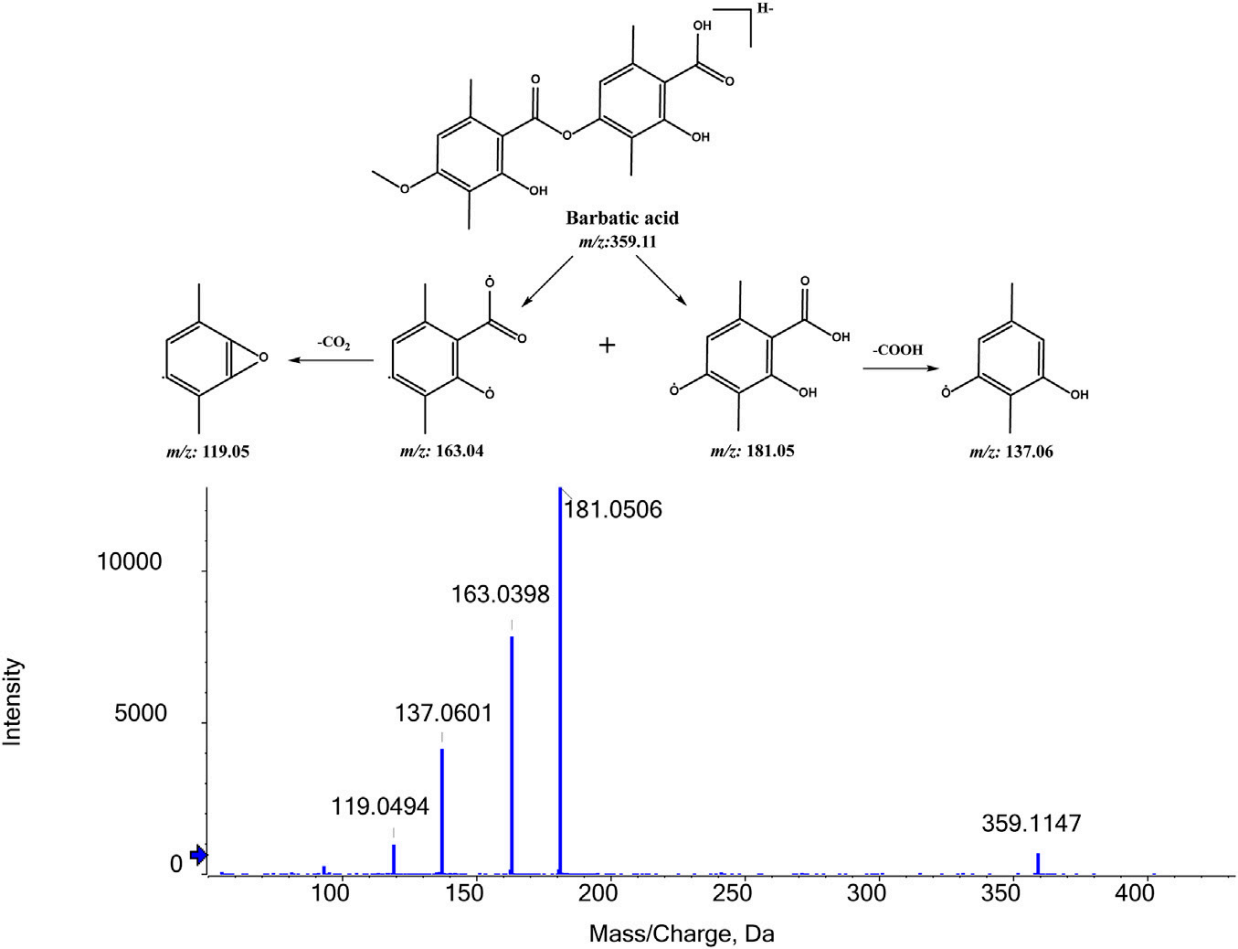
MS/MS spectrum of BA and its proposed fragmentation pathways.

To identify the metabolites of BA, the MS/MS fragmentation behaviors of BA were first investigated by UPLC/ESI-QTOF-MS. BA was eluted at 9.354 min and showed [M-H]^-^ at m/z 359.1147 (C_19_H_19_O_7_^−^, 6.0 ppm). BA provided abundant fragment ions at [M-H-C_10_H_10_O_3_]^-^ m/z 181.0506 (C_9_H_9_O_4_^−^, 5.9 ppm) [M-H-C_10_H_12_O_4_]^-^ m/z 163.0390 (C_9_H_7_O_3_^−^, 5.1 ppm) [M-H-C_10_H_10_O_3_-COO]^-^ m/z 137.0601 (C_8_H_9_O_2_^−^, 2.9 ppm), and [M-H-C_10_H_12_O_4_-COO]^-^ m/z 119.0494 (C_8_H_7_O^−^, 2.2 ppm). Figure 7 shows the proposed fragmentation pathways of BA.

#### 3.2.2 Metabolites of hydroxylation (m/z 375.1074)

Metabolite **M2-1** showed a quasi-molecular ion at m/z 375.1109([M-H]^-^, C_19_H_19_O_8_^−^, 9.2 ppm) and could be detected at 6.832 min.The MS/MS spectra presented fragment ions at [M-H-C_10_H_10_O_4_]^-^ m/z 181.0514 (C_9_H_9_O_4_^−^, 10.3 ppm) [M-H-C_10_H_12_O_5_]^-^ m/z 163.0411 (C_9_H_7_O_3_^−^, 13.1 ppm) [M-H-C_10_H_10_O_4_-COO]^-^ m/z 137.0614 (C_8_H_9_O_2_^−^, 12.4 ppm), and [M-H-C_10_H_12_O_5_-COO]^-^ m/z 119.0503 (C_8_H_7_O^−^, 9.7 ppm). The proposed fragmentation pathway of metabolites **M2-1** was similar to that of BA. As no more information was observed, the specific structure could not be established from the mass spectrum data alone. Supplementary Figure S9 presents MS/MS spectra and the proposed fragmentation pathway of **M2-1**.

#### 3.2.3 Metabolites of demethylation (m/z 389.1242)

Metabolite **M2-2** presented a quasi-molecular ion at m/z 389.1260 ([M-H]^-^, C_20_H_21_O_8_^−^, 2.8 ppm) and could be detected at 6.436 min.The MS/MS spectra exhibited fragment ions at [M-H-C_11_H_12_O_4_]^-^ m/z 181.0515 (C_9_H_9_O_4_^−^, 10.9 ppm) [M-H-C_11_H_14_O_5_]^-^ m/z 163.0394 (C_9_H_7_O_3_^−^, 2.6 ppm) [M-H-C_11_H_12_O_4_-COO]^-^ m/z 137.0602 (C_8_H_9_O_2_^−^, 3.6 ppm), and [M-H-C_11_H_14_O_5_-COO]^-^ m/z 119.0512 (C_8_H_9_O_2_^−^, 17.3 ppm). The proposed fragmentation pathway of metabolite **M2-2** was similar to that of BA. As no more information was observed, the specific structure could not be established from the mass spectrum data alone. Supplementary Figure S10 shows the MS/MS spectra and the proposed fragmentation pathway of **M2-2**.

#### 3.2.4 Metabolites of hydroxylation and methylation (m/z 389.1242)

Metabolite **M2-3** showed the quasi-molecular ion at m/z 389.1260 ([M-H]^-^, C_20_H_21_O_8_^−^, 2.8 ppm) and could be detected at 6.436 min.The MS/MS spectra revealed fragment ions at [M-H-COO]^-^ m/z 345.1334 (C_19_H_21_O_6_^−^, 0.4 ppm) [M-H-COOH-CH_2_-OH]^-^ m/z 313.1101 (C_18_H_17_O_5_^−^, 9.7 ppm) [M-H-COOH-CH_2_-OH-CH_3_]^-^ m/z 298.0836 (C_17_H_14_O_5_^−^, 0.1 ppm) [M-H-COO-CH_3_-CH_2_-2OH]^-^ m/z 283.0972 (C_17_H_15_O_4_^−^, 2.5 ppm) [M-H-COO-2CH_3_-CH_2_-2OH]^-^ m/z 268.0761 (C_16_H_12_O_4_^−^, 11.5 ppm) [M-H-C_10_H_8_O_5_]^-^ m/z 181.0854 (C_10_H_13_O_3_^−^, −2.9 ppm) [M-H-C_10_H_8_O_5_-CH_2_O]^-^ m/z 151.0773 (C_9_H_11_O_2_^−^, 12.9 ppm), and [M-H-C_10_H_8_O_5_-2CH_2_O]^-^ m/z 121.0658 (C_8_H_9_O^−^, 8.3 ppm). Supplementary Figure S11 exhibits the MS/MS spectra and the proposed fragmentation pathway of **M2-3**.

#### 3.2.5 Metabolites of demethylation and carbonylation (m/z 359.0772)

Metabolite **M2-4** presented a quasi-molecular ion at m/z 359.0786 ([M-H]^-^, C_18_H_15_O_8_^−^, 6.8 ppm) and could be detected at 6.688 min.The MS/MS spectra displayed fragment ions at [M-H-OH-H]^-^ m/z 341.0671 (C_18_H_13_O_7_^−^, 4.5 ppm) [M-H-CHO-OH]^-^ m/z 313.0701 (C_17_H_13_O_6_^−^, −1.8 ppm) [M-H-COOH-OH]^-^ m/z 297.0765 (C_17_H_13_O_5_^−^, 2.5 ppm), and [M-H-COOH-CHO-CH_3_]^-^ m/z 270.0519 (C_15_H_10_O_5_^−^, −1.3 ppm). As no more information was observed, the specific structure could not be established from the mass spectrum data alone. Supplementary Figure S12 includes the MS/MS spectra and the proposed fragmentation pathway of **M2-4**.

#### 3.2.6 Metabolites of hydroxylation and demethylation (m/z 361.0929)

Metabolite **M2-5** manifested the quasi-molecular ion at m/z 361.0904 ([M-H]^-^, C_18_H_17_O_8_^−^, −3.9 ppm) and could be detected at 6.248 min.The MS/MS spectra presented fragment ions at [M-H-C_9_H_8_O_3_]^-^ m/z 197.0484 (C_9_H_9_O_5_^−^, 20.0 ppm) [M-H-C_9_H_8_O_4_]^-^ m/z 181.0495 (C_9_H_9_O_4_^−^, −0.2 ppm) [M-H-C_9_H_10_O_5_]^-^ m/z 163.0411 (C_9_H_7_O_3_^−^, 2.0 ppm) [M-H-C_9_H_8_O_4_-COO]^-^ m/z 137.0604 (C_8_H_9_O_2_^−^, 5.1 ppm), and [M-H-C_9_H_10_O_5_-COO]^-^ m/z 119.0492 (C_8_H_7_O^−^, 0.5 ppm). The proposed fragmentation pathway of metabolite **M2-5** was similar to that of BA. As no more information was observed, the specific structure could not be established from the mass spectrum data alone. Supplementary Figure S13 contains the MS/MS spectra and the proposed fragmentation pathway of **M2-5**.

#### 3.2.7 Metabolites of glucuronidation (m/z 535.1452)

Five glucuronidation metabolites of BA were identified in the microsomal incubation system *in vitro*. Metabolites **M2-6** (t_R_ = 5.388 min), **M2-7** (t_R_ = 6.064 min), and **M2-8** (t_R_ = 7.173 min) showed the same quasi-molecular of C_25_H_27_O_13_^−^ (m/z 535.1452 [M-H]^-^), which was 176.0321 Da higher than that of BA m/z 359.1147 (C_19_H_19_O_7_^−^, 6.0 ppm). This finding indicated the characteristic loss of the glucuronic acid group (C_6_H_10_O_7_-H_2_O). In the MS/MS spectra, the [M-H]^-^ of **M2-6** showed fragment ions at [M-H-C_6_H_8_O_6_]^-^ m/z 359.1139 (C_19_H_19_O_7_^−^, 3.8 ppm) [M-H-C_6_H_8_O_6_-COO]^-^ m/z 315.1228 (C_18_H_19_O_5_^−^, 0.3 ppm) [M-H-C_6_H_8_O_6_-C_10_H_10_O_3_]^-^ m/z 181.0511 (C_9_H_9_O_4_^−^, 8.6 ppm) [M-H-C_6_H_8_O_6_-C_10_H_12_O_4_]^-^ m/z 163.0404 (C_9_H_7_O_3_^−^, 8.8 ppm) [M-H-C_6_H_8_O_6_-C_10_H_10_O_3_-COO]^-^ m/z 137.0610 (C_8_H_9_O_2_^−^, 9.4 ppm), and [M-H-C_20_H_22_O_10_]^-^ m/z 113.0241 (C_5_H_5_O_3_^−^, 6.9 ppm). The [M-H]^-^ of **M2-7** presented fragment ions at [M-H-C_6_H_8_O_6_]^-^ m/z 359.1136 (C_19_H_19_O_7_^−^, 3.0 ppm) [M-H-C_6_H_8_O_6_-C_10_H_10_O_3_]^-^ m/z 181.0512 (C_9_H_9_O_4_^−^, 9.2 ppm) [M-H-C_6_H_8_O_6_-C_10_H_12_O_4_]^-^ m/z 163.0405 (C_9_H_7_O_3_^−^, 9.4 ppm) [M-H-C_6_H_8_O_6_-C_10_H_10_O_3_-COO]^-^ m/z 137.0608 (C_8_H_9_O_2_^−^, 8.0 ppm), and [M-H-C_20_H_22_O_10_]^-^ m/z 113.0241 (C_5_H_5_O_3_^−^, 16.6 ppm). The [M-H]^-^ of **M2-8** exhibited fragment ions at [M-H-C_6_H_8_O_6_]^-^ m/z 359.1099 (C_19_H_19_O_7_^−^, −7.3 ppm) [M-H-C_6_H_8_O_6_-C_10_H_10_O_3_]^-^ m/z 181.0495 (C_9_H_9_O_4_^−^, −0.2 ppm), and [M-H-C_6_H_8_O_6_-C_10_H_12_O_4_]^-^ m/z 163.0419 (C_9_H_7_O_3_^−^, 18.0 ppm). The proposed fragmentation pathway of metabolites **M2-6** to **M2-8** was similar to that of BA. Moreover, the fragmentation behaviors between these metabolites were very similar. As no more information was observed, the specific structure could not be established from the mass spectrum data alone. Supplementary Figure S14 summarizes the MS/MS spectra and the proposed fragmentation pathway of the glucuronidation metabolites.

### 3.3 CYP phenotyping reaction

Chemical inhibition studies reported the different effects of CYP inhibitors on the biotransformation of UA. CYP3A4, 1A1, and 2C9 showed low inhibition effects on **M1-1,** and CYP2A6 exhibited a weak inhibition effect only at 5 µM with RLMs. CYP2B6 presented a strong inhibitory effect on **M1-3,** followed by CYP1A1, 2A6, 2C9, 2C19, and 3A4 with RLMs. As for **M1-4**, CYP2B6, 2A6, 1A1, 3A4, 2C9, 2C19, 2D6, 2E1,1A2, and 2C8 showed high to low levels of inhibition effect with RLMs. CYP2B6 and 1A1 with RLMs showed a stronger inhibition effect on **M1-6** than CYP3A4, 2D6, 2C9, 2C19, 2A6, 1A2, and 2C8. CYP2B6 and 1A1 with RLMs also displayed a stronger inhibition effect on **M1-7**. In addition, CYP3A4, 2A6, 2C9, 2D6, 2C19, 2C8, 1A2, and 2E1 exhibited high to low levels of inhibition effect with RLMs. As for **M1-8**, only CYP2C8, 2C19, and 1A1 with RLMs showed low inhibition effects. CYP2C19, 2C8, and 2B6 with RLMs revealed a weak inhibition effect on **M1-9**. For **M1-10**, CYP2B6, 1A1, 2A6, and 2C9 presented high to low levels of inhibition effect with RLMs, and CYP3A4 and 2C19 with RLMs showed weak inhibition effects only at 100 µM. For **M1-11**, CYP2B6, 2A6, 1A1, 2C9, 2C19, 3A4, 2D6, 2E1, and 1A2 with RLMs showed high to low levels of inhibition effects.

**FIGURE 8:**
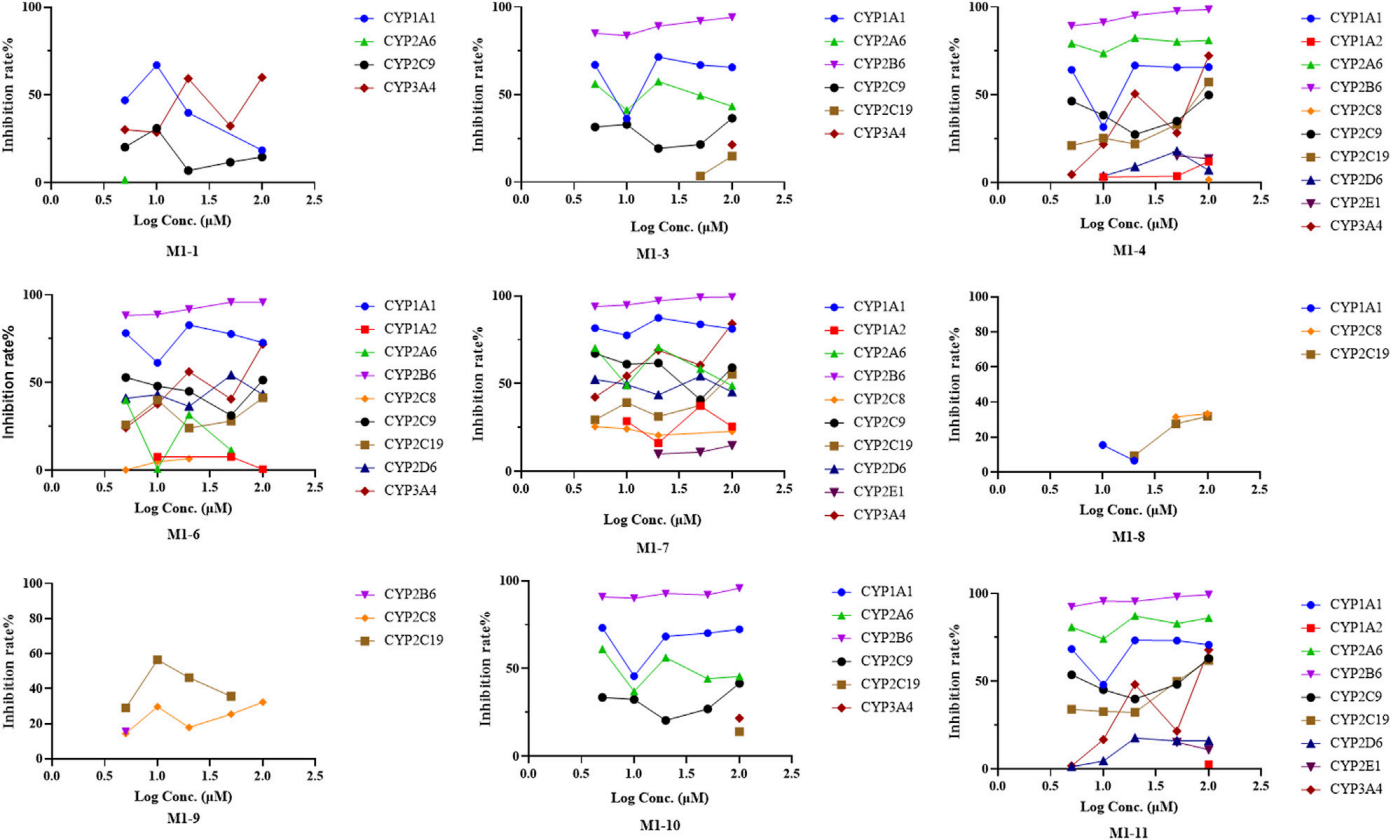
Inhibition rate of UA metabolites in RLM in different chemical inhibitors treatment.

With HLMs, CYP3A4, 2E1, 2B6, 2C19, 2D6,1A2, 2C8, 1A1, and 2A6 presented high to low levels of inhibition effects on **M1-1**. CYP1A1 and 2A6 showed weak inhibition effects only at 5 µM. For **M1-3**, CYP2C9, 2E1, 2D6, 2B6, 2C19, 2C8, 3A4, 1A2, 1A1, and 2A6 with HLMs displayed high to low levels of inhibition effects. For **M1-6**, CYP2C9, 2E1, 2D6, 2B6, 1A1, 2C19, 1A2, and 2A6 with HLMs exhibited high to low levels of inhibition effects, and CYP2C19 with HLMs showed weak inhibition effect only at 5 µM. For **M1-8,** CYP3A4, 2E1, 2C9, 2B6, 2C19, 1A2, 2C8, and 2D6 with HLMs manifested high to low levels of inhibition effects, and CYP2A6 and 1A1 with HLMs showed a mild inhibition effect only at 5 µM. For **M1-10**, CYP2C9 presented a strong inhibition effect at all concentrations with HLMs. In addition, CYP2E1, 2D6, 2B6, 1A2, 1A1, 3A4, 2C19, 2C8, and 2A6 showed moderate inhibition effects with HLMs. For **M1-13**, CYP2E1, 2D6, 2B6, 3A4, 2C8, 2C19, 2A6, 1A2, 1A1, and 2C9 presented high to low levels of inhibition effects with HLMs. For **M1-14**, CYP2E1, 2D6, 3A4, 2C19, 2B6, 2C9, 2C8, 2A6, 1A1, and 1A2 showed moderately high to low inhibition effects with HLMs. Compared with the control group, the inhibitors of CYP2B6, 1A1, and 2A6 with RLMs inhibited UA metabolism the most (Figure 8), and CYP2C9 and 2E1 inhibitors with HLMs presented the strongest inhibitory effect on UA metabolism (Supplementary Figure S15).

**FIGURE 9:**
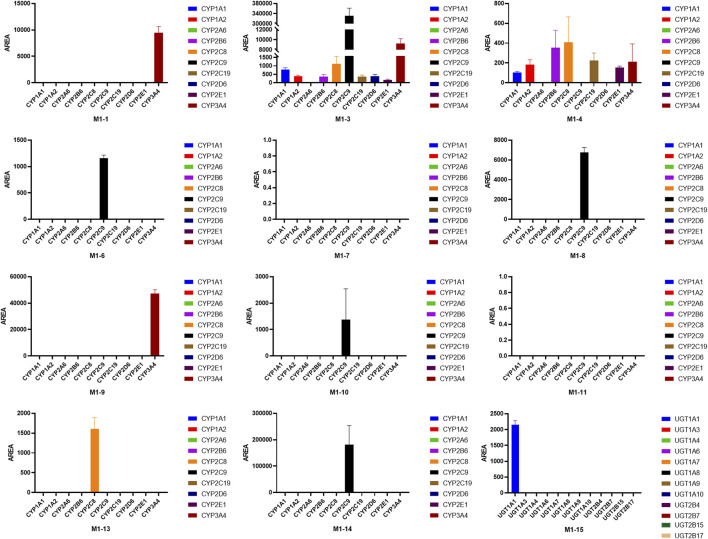
Peak areas of UA metabolites in different recombinant human CYP and UGT isozymes.

As shown in Figure 9, the main isozymes tested for UA metabolism were CYP2C9, 3A4, and 2C8. Metabolites **M1-1**, **M1-4**, and **M1-9** were mainly metabolized by CYP3A4, and metabolites **M1-3**, **M1-6**, **M1-8**, **M1-10**, and **M1-14** were mainly metabolized by CYP2C9. Moreover, metabolites **M1-4** and **M1-13** were mainly metabolized by CYP2C8. Only **M1-3** and **M1-4** were metabolized by multiple metabolic enzymes, and CYP2A6 was the only isozyme that showed no contribution to the metabolism of UA. UGT1A1 was the only isozyme involved in phase II metabolite **M1-15** production. Metabolites **M1-2, M1-5**, and **M1-12** were NADPH-independent products. The production of **M1-7**, **M1-11**, **M1-16**, **M1-17**, and **M1-18** was not associated with the tested recombinant CYP and UGT enzymes, and their generation was speculated to be related to other factors.

**FIGURE 10:**
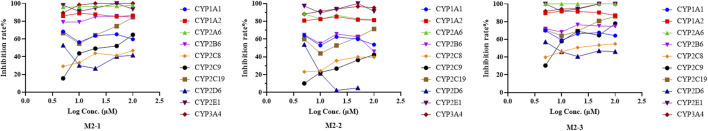
Inhibition rate of BA metabolites in RLM in different chemical inhibitors treatment.

For metabolite **M2-1**, CYP3A4, 2E1, 2A6, 1A2, 2B6, 2C19, 1A1, 2C9, 2C8, and 2D6 with RLMs presented high to low levels of inhibition effects. For metabolite **M2-2**, CYP3A4, 2E1, 2A6, and 1A2 with RLMs showed a higher inhibition effect than CYP2B6, 1A1, 2C19, 2C8, 2C9, and 2D6. For metabolite **M2-3**, CYP2A6, 3A4, 2E1, 1A2, 2B6, 2C19, 1A1, 2C9, 2C8, and 2D6 with RLMs displayed relatively high inhibition effects. With HLMs, only CYP2A6 and 3A4 revealed a weak inhibition effect on **M2-1**. For metabolite **M2-2**, CYP2A6, 1A1, 1A2, 3A4, and 2C8 with HLMs revealed weak inhibition effects, whereas CYP2B6, 2C9, and 2E1 presented a low inhibition effect at 10, 5, and 50 and 100 μM, respectively. For metabolite **M2-4**, CYP2A6 showed an inhibition effect from 20 µM to 100 μM, and CYP3A4 presented an inhibition effect only at 100 µM. For metabolite **M2-5**, only CYP3A4 manifested an inhibition effect at 100 µM. Compared with the control group, CYP2E1, 3A4, 2A6, and 1A2 with RLMs displayed a more evident inhibitory effect on the metabolites of BA (Figure 10). However, the inhibition rate of BA metabolites in HLMs was unclear (Supplementary Figure S16).

**FIGURE 11:**
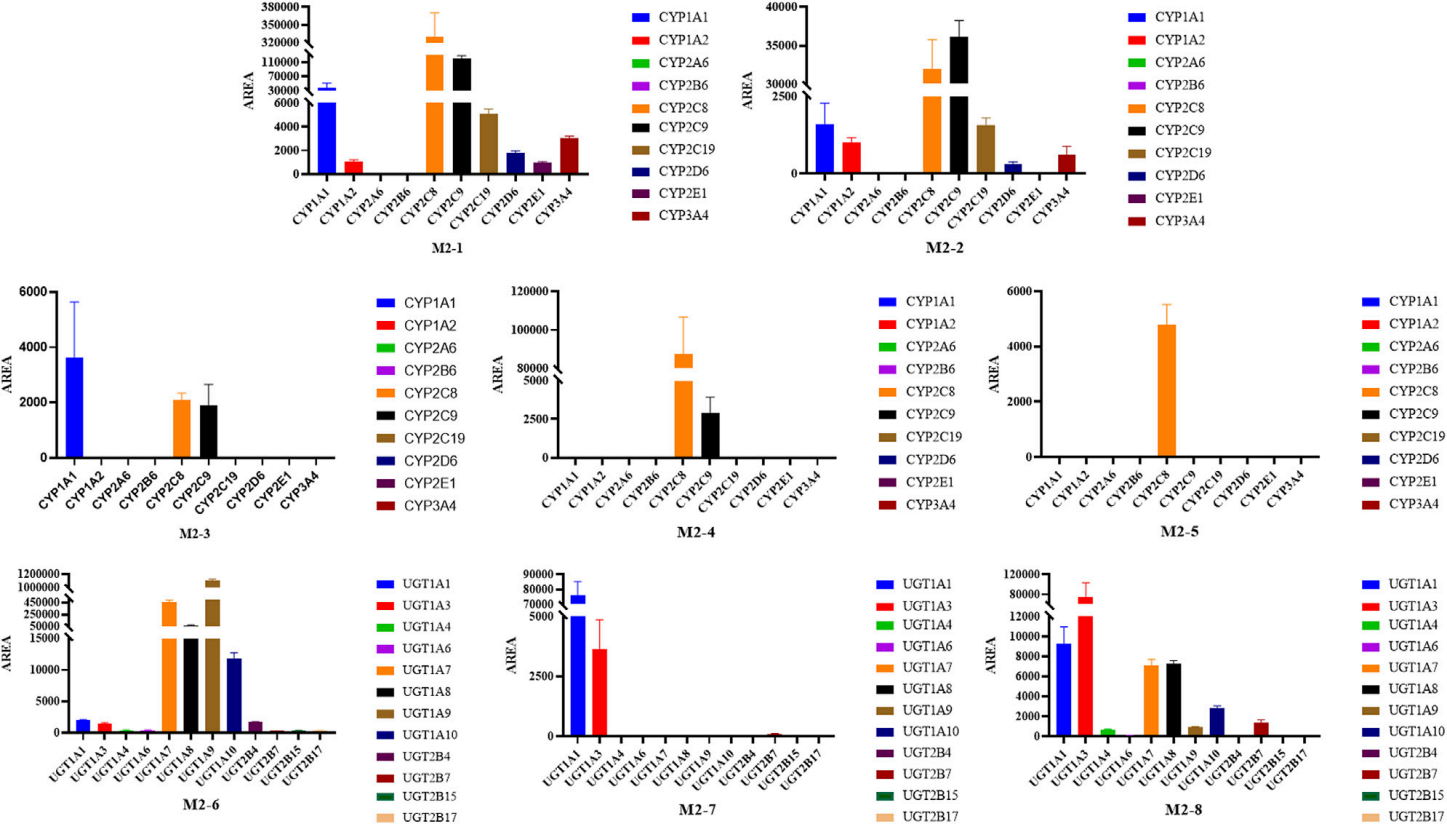
Peak areas of BA metabolites in different recombinant human CYP and UGT isozymes.

Most tested isozymes were involved in the generation of BA metabolites (Figure 11), which differed from that of UA. CYP2C8 played an important role in the generation of all phase I metabolites of BA from **M2-1** to **M2-5** and was the major isozyme involved in the metabolism of **M2-1**, **M2-4**, and **M2-5**. CYP2C9 has been implicated in the metabolism of **M2-1** to **M2-4** and is one of the major isozymes of **M2-1** and **M2-2**. CYP1A1 was also involved in the generation of **M2-1**, **M2-2**, and **M2-3**. In addition, most of the tested isozymes were involved in the generation of **M2-1** and **M2-2**, with CYP2C8, 2C9, 1A1, 2C19, 1A2, 3A4, 2D6, and 2E1 producing metabolites in descending order. Although the participation of several isozymes was inevident, the generation of **M2-6** involved all the tested isozymes. UGT1A9, 1A7, 1A8, and 1A10 were the major isozymes responsible for the phase II metabolism of BA. **M2-7** was mainly metabolized by UGT1A1, 1A3, and several 2B7. The generation of **M2-8** was mainly involved in UGT1A3, 1A1, 1A7, 1A8, and 1A10 with several 2B7, 1A9, and 1A4.

### 3.4 Cytotoxicity assay

**FIGURE 12:**
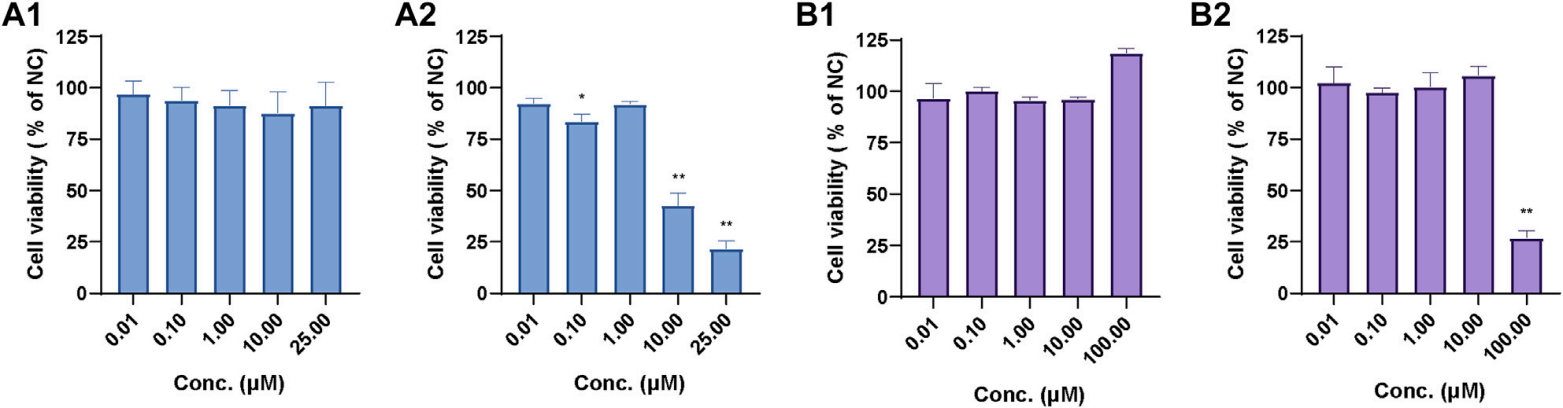
Relative cell viability of UA in human primary hepatocytes **(A1)** and mouse 3T3 fibroblasts **(A2)** and relative cell viability of BA in human primary hepatocytes **(B1)** and mouse 3T3 fibroblasts **(B2)**. Data are expressed as mean ± SD, *n* = 3, **p < 0.01, *p < 0.05 vs. NC group.

As shown in Figure 12A1, the relative cell viability (% of normal control, NC) of 0.01 µM–25 µM UA in human primary hepatocytes ranged from 87.48% to 96.65%, which indicates that UA had no cytotoxicity toward human primary hepatocytes within the test range. Meanwhile, the relative cell viability (% of NC) of 0.01 µM–1 µM UA in mouse 3T3 fibroblasts (Figure 12A2) ranged from 83.30% to 92.01%. The relative cell viability decreased to 42.58% and 21.52% at 10 and 100 μM, respectively. This result indicates that UA did not exhibit cytotoxicity against mouse 3T3 fibroblasts at low concentrations. UA was cytotoxic at a higher concentration range, and its cytotoxicity increased with the increase in concentrations.

The relative cell viability (% of NC) of 0.01 µM–100.00 µM BA in human primary hepatocytes (Figure 12B1) ranged from 95.72% to 118.75%. The relative cell viability of 0.01 µM–10.0 µM BA in mouse 3T3 fibroblasts (Figure 12B2) ranged from 97.56% to 106.05% and decreased to 26.87% at 100.00 µM, which suggests that BA exhibited cytotoxicity against mouse 3T3 fibroblasts at 100.00 µM.

### 3.5 Effect of metabolism on cytotoxicity

**FIGURE 13:**
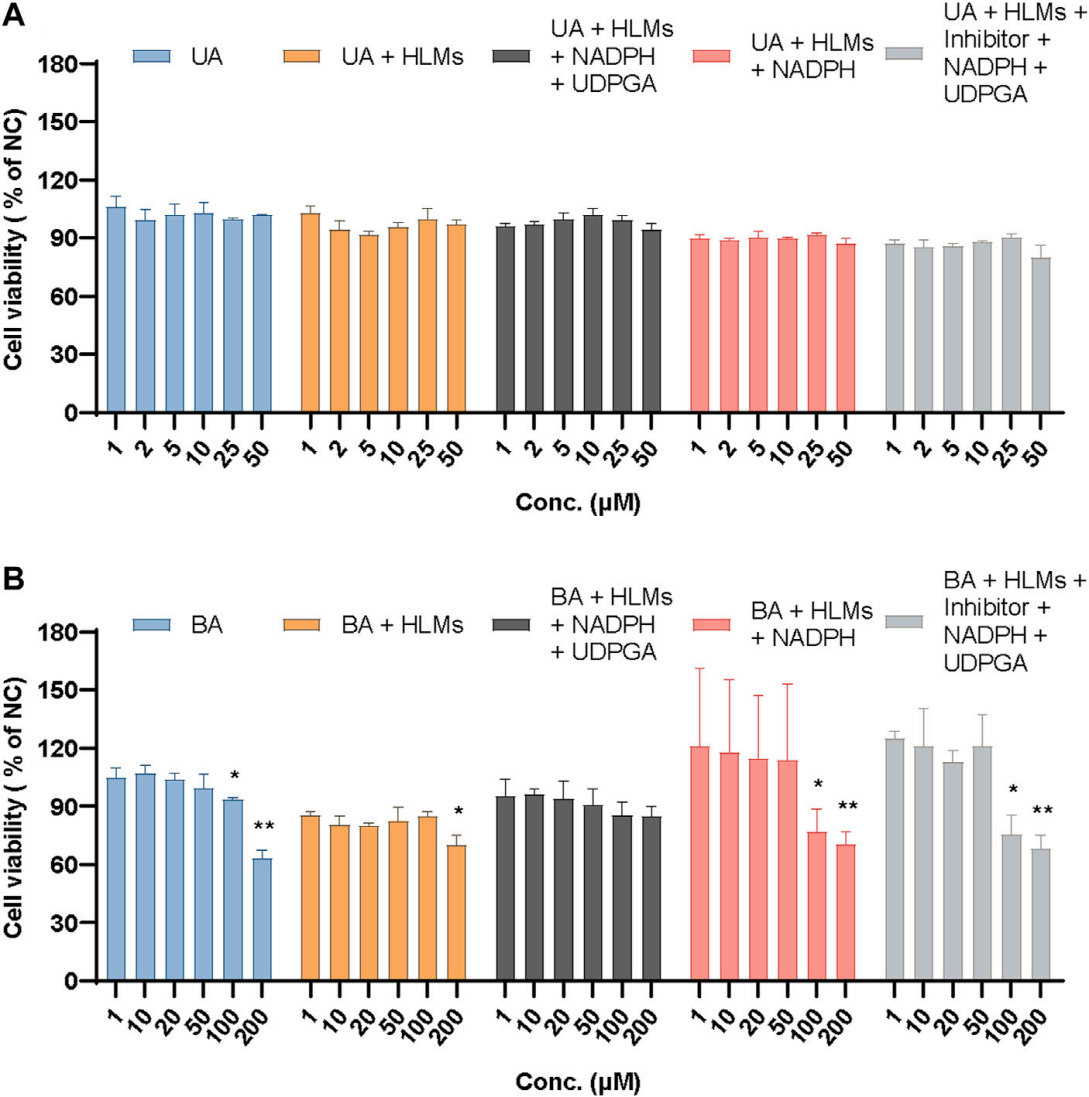
Relative cell viability of UA **(A)** and BA **(B)** in mouse 3T3 fibroblasts in different incubation groups. Data are expressed as mean ± SD, *n* = 3, **p < 0.01, *p < 0.05 vs. NC group.

To confirm the contribution of UA and BA or their metabolites to the cytotoxicity of mouse 3T3 fibroblasts, we studied the effects of UA or BA incubation with HLMs, NADPH and UDPGA, and montelukast, sulfaphenazole, and ketoconazole, the exclusive inhibitors of CYP2C8, CYP2C9, and CYP3A4, by measuring cell viability. After incubation with mouse 3T3 fibroblasts for 4 h, the cell viability of UA groups from 1 μM to 50 µM ranged from 80.04% to 106.16%. None of the five groups exhibited cytotoxicity. The results showed that the cytotoxicity mechanism of UA may be related to long-term toxicity as it did not show cytotoxicity within 4 h of incubation in each group (Figure 13A).

Based on previous experiments, the relative cell viability showed that 100 µM BA exhibited cytotoxicity after incubation with mouse 3T3 fibroblasts for 24 h (26.87%) but not after 4 h (93.35%). Meanwhile, 200 µM BA displayed cytotoxicity after incubation with mouse 3T3 fibroblasts for 4 h (63.07%), which indicated that the cytotoxicity of BA increased in a time- and concentration-dependent manner. When incubated with HLMs, 20 and 200 µM BA showed cytotoxicity toward mouse 3T3 fibroblasts (79.80% and 69.73%, respectively). The relative cell viability of these groups had an overall downward trend from 85.27% to 69.73%. After incubation with HLMs and NADPH or HLMs, NADPH, UDPGA, and inhibitors of CYP2C8, CYP2C9, and CYP3A4 for 4 h, the relative cell viability (% of NC) of 100 and 200 μM BA, which exhibited cytotoxicity toward mouse 3T3 fibroblasts, ranged from 68.26% to 76.84%. BA showed no cytotoxicity to mouse 3T3 fibroblasts with HLMs, NADPH, and UDPGA only (Figure 13B). These results suggest that the cytotoxicity of BA originates from itself, which suggests a metabolic detoxification mechanism, and that UGTs may act as the main metabolic detoxification enzyme. Supplementary Table S1 shows the data of positive control and mix inhibitors.

## 4 Discussion

Drug-induced liver injury is one of the most common liver diseases. As the earliest lichen compound has been commercialized and developed, recent reports associated with liver-related adverse events of UA and other lichen compounds contained in products aroused widespread concern. Furthermore, the toxicity and mechanism of UA and other lichen compounds that induce hepatotoxicity remain unclear and have become a highly valued topic.

Thus far, conflicting results and reports have been reported regarding UA toxicity, and the reasons for the hepatotoxicity caused by UA have not been determined. For example, several reports described the hepatotoxicity of UA from a dietary supplement Lipokinetix^®^, which is composed of norephedrine hydrochloride (25 mg), sodium usniate (100 mg), 3,5-diiodothyronine (100 μg), yohimbine hydrochloride (3 mg), and caffeine (100 mg) (Favreau et al., 2002; Durazo et al., 2004; Sanchez et al., 2006; Stickel et al., 2009; Yellapu et al., 2011; Avigan et al., 2016; Brown, 2017). In addition to its effect on humans, it was reported that UA can cause moderate hepatic injury in a dose-dependent manner in rats. Studies showed no hepatotoxicity in male Wistar albino rats after oral administration of UA at doses of 500 and 1000 mg/kg. However, hepatotoxicity was observed at higher doses of UA, such as 2000 mg/kg. Thus, UA-induced hepatotoxicity is dose-dependent and occurs only at a certain concentration. This could be expected since most compounds, in general, increase their chances of becoming potentially toxic to the body at high doses over long periods of consumption (Odabasoglu et al., 2006; Guo et al., 2008; Lu et al., 2011; Liu et al., 2012). The toxicity and potential harm of potentially toxic substances should not be underestimated. The dose-dependent toxicity of UA is directly related to the process of ADME (absorption, distribution, metabolism, and elimination) *in vivo* and systemic exposure level, among which, in our opinion, drug metabolism is one of the key factors affecting the exposure level.

Most cellular studies showed that UA can cause cell necrosis and affects mitochondrial function (Araujo et al., 2015). Early studies showed that UA is an uncoupler of oxidative phosphorylation in mouse liver mitochondria (Abo-Khatwa et al., 1996; Pramyothin et al., 2004). The uncoupling activity in isolated rat liver mitochondria has been detected with 0.15 µM–6 μM UA, whereas the loss of cell membrane integrity in isolated rat hepatocytes can be induced by increasing the intracellular release of aspartate transaminase and alanine transaminase with a high dose UA (1 mM). In addition, the activities of cell lipid peroxidation and aniline hydroxylase increased, and the content of GSH decreased with the high dose of UA (Abo-Khatwa et al., 1996; Pramyothin et al., 2004). We speculated that the hepatotoxic effect of a high dose of UA may involve its reactive metabolites.

Currently, several scholars suggest that when CYP1A is inhibited, the metabolism of UA will decrease and result in its accumulation, which leads to the excessive inhibition of mitochondrial respiration, insufficient ATP, and cell necrosis. Therefore, several CYP enzyme inhibitors may increase the cytotoxicity of UA on rat hepatocytes, implying that UA might have a metabolic detoxification mechanism (Shi et al., 2014). Another research reported that UA can reduce GSH in hepatocytes and inhibit the synthesis of ATP in mitochondria, which leads to cell oncosis but not cell necrosis or apoptosis (Kwong et al., 2020).

In this study, the metabolic profiles of UA and BA were investigated in RLMs, HLMs, and RS9. A total of 14 phase I metabolites and 4 phase II metabolites of UA and 4 phase I metabolites and 6 phase II metabolites of BA were identified. The results revealed that the metabolism of UA and BA with RLMs and HLMs was induced by CYP450 or UGTs and mediated by hydroxylation, methylation, and glucuronidation reactions. These metabolites showed similar fragmentation patterns in MS/MS spectra and only differed in fragment abundance.

UA is metabolized primarily by CYP1A2, UGT1A1, UGT1A3, and UGT1A8 (Foti et al., 2008). In addition, UA is a potent inhibitor of CYP2C19 and CYP2C9, a significantly weak inhibitor of CYP2C8 and CYP2C18, and a relatively weak inhibitor of CYP2D6. Another study used 20 mM SKF-525A (a non-isoenzyme-selective inhibitor), 10 mM alpha-naphthoflavone (a CYP1A inhibitor), and 25 mM ketoconazole (CYP3A inhibitor) to verify whether UA metabolism in rat primary hepatocytes leads to the formation of cytotoxic metabolites or whether metabolism is a detoxification process. The metabolites of UA were less toxic in rat primary hepatocytes and probably transformed mainly by CYP1A and 3A but not 2B/2C (Shi et al., 2014). The present result suggests that only the metabolites of **M1-2** and **M1-3** were partially metabolized by CYP1A2. The metabolites of **M1-1**, **M1-3**, **M1-4**, and **M1-9** were mainly influenced by CYP3A4, and **M1-15** was regulated with UGT1A1. CYP2C9, CYP3A4, CYP2C8, and UGT1A1 are the main metabolic enzyme subtypes responsible for several metabolites of UA.

It is worth mentioning that there are no studies on enzymes responsible for BA metabolism *in vivo* and *in vitro*. Therefore, this study presents novel results that indicate that CYP2C8, CYP2C9, CYP2C19, CYP1A1, UGT1A1, UGT1A3, UGT1A7, UGT1A8, UGT1A9, and UGT1A10 are the main metabolic enzymes responsible for several metabolites of BA.

The identification of metabolic enzymes is a challenging study and is influenced by numerous factors and research strategies, such as the selection of target metabolites, activity of metabolic enzymes, specificity and inhibitory intensity of inhibitors, co-participation of multiple metabolic enzymes, and composition of the incubation system. In general, the results confirmed by recombinant isozymes are more reliable. To determine the major metabolic enzymes responsible for a compound, scholars must first identify the major metabolites from multi-metabolites or identify the metabolites related to metabolic activation or metabolic detoxification and then determine metabolic enzymes based on specific metabolites. However, only metabolites and the metabolic pathways of UA and BA were identified and analyzed in this research paper. Given the lack of separation, preparation, and structure confirmation of metabolites, quantitative determination of metabolites is impossible, which leads to the failure of identifying major and key metabolites based on existing results. Therefore, the identification of the key metabolic enzymes responsible for UA and BA is extremely difficult. This work will be further carried out in follow-up studies.

The cytotoxic mechanism of drugs is usually based on their effect on hepatic drug-metabolizing enzymes (Liu et al., 2010), such as pyrrolizidine alkaloids senecionine (Williams et al., 1989), isoline (Tang et al., 2007), diosbulbin B (Jiang et al., 2017), and neferine (Shen et al., 2014). The cytotoxicity and metabolic toxicity mechanism of UA and BA were studied by human primary hepatocytes and mouse 3T3 fibroblasts. UA and BA showed no significant effect on the cell activity of human primary hepatocytes at concentrations of 0.01–25 and 0.01–100 μM, respectively, but had a significant inhibitory effect on the cell activity of mouse 3T3 fibroblasts with IC_50_ values of 7.40 and 60.2 µm. The cytotoxicity of UA may be associated with chronic toxicity. The cytotoxicity of BA is associated with metabolism, and UGTs may be the main metabolic detoxification enzymes.

All health benefits or harmful effects (side effects) of *Usnea* are due to the coexisting compounds contained in the lichen. Considering a mixture extract of plant herb or polyherbal compound, drug-drug interaction between UA and BA or other coexisting components must be taken into account. In particular, attention should be paid to the inhibitory and inductive effects of different compounds on drug-metabolizing enzymes and on transporters that play key roles in absorption and excretion processes (Truong et al., 2021).

Despite the difficulty of determining the exact structure of metabolites through MS only and confirming the specific mechanism of metabolic detoxification completely through *in vitro* experiments, the present results provide important insights into the understanding of the biotransformation behavior and metabolic detoxification of UA and BA. They will provide important references for the research of metabolism behavior and clinical pharmacology of UA, BA, and other similar lichen compounds *in vivo.*

## 5 Conclusion

In conclusion, hydroxylation, methylation, and glucuronidation reactions were involved in the metabolic profiles of UA and BA in RLMs, HLMs, and RS9 mediated by CYP450 and UGT. CYP2C9, CYP3A4, CYP2C8, and UGT1A1 were the key metabolic enzymes responsible for the metabolism of UA. CYP2C8, CYP2C9, CYP2C19, CYP1A1, UGT1A1, UGT1A3, UGT1A7, UGT1A8, UGT1A9, and UGT1A10 were the main metabolic enzymes responsible for the metabolism of BA. UA and BA did not display evident cytotoxicity toward human primary hepatocytes but did so toward mouse 3T3 fibroblasts. The attenuated cytotoxicity of BA was associated with metabolism, and UGTs may be the key metabolic detoxification enzymes. The cytotoxicity of UA may be associated with chronic toxicity. Overdose and long-term consumption of UA and BA, as well as the medicinal materials containing these compounds, still have potential risks of liver toxicity, and their application and safety deserve to be studied and receive more attention.

## Data Availability

The datasets presented in this study can be found in online repositories. The names of the repository/repositories and accession number(s) can be found in the article/Supplementary Material.
