# Supplementary material for: Metabolism and toxicity of usnic acid and barbatic acid based on microsomes, S9 fraction, and 3T3 fibroblasts in vitro combined with a UPLC-Q-TOF-MS method
Source: Front Pharmacol. 2023 Jun 15;14:1207928. doi: 10.3389/fphar.2023.1207928 (PMC10308081; doi:10.3389/fphar.2023.1207928)
Supplement: Supplementary file 1 [file DataSheet1.DOCX]

Supplementary Material

Metabolism and Toxicity of Usnic Acid and Barbatic Acid Based on Microsomes, S9 Fraction, and 3T3 Fibroblasts *in vitro* Combined with UPLC-Q-TOF-MS

Hanxue Wang^1,2,3^, Min Xuan^4^, Juanjuan Diao^5^, Nan Xu^3^, Manlin Li^3^, Cheng Huang^2,^ *, and Changhong Wang^3,^ *

^1^Shanghai TCM-Integrated Hospital, Shanghai University of Traditional Chinese Medicine, Shanghai, China

^2^School of Pharmacy, Shanghai University of Traditional Chinese Medicine, Shanghai, China

^3^The MOE Key Laboratory for Standardization of Chinese Medicines and The SATCM Key Laboratory for New Resources and Quality Evaluation of Chinese Medicine, Shanghai Key Laboratory for TCM Complex Prescription, Institute of Chinese Materia Medica, Shanghai University of Traditional Chinese Medicine, Shanghai, China

^4^Department of Pharmacy, Qingdao Eighth People’s Hospital, Qingdao, China

^5^Analysis and Testing Center, Xinjiang Medical University (Xuelanshan Campus), Urumqi, China

***Correspondence:** Cheng Huang: [chuang@shutcm.edu.cn](mailto:chuang@shutcm.edu.cn), Changhong Wang: [wchcxm@hotmail.com](mailto:wchcxm@hotmail.com); [wchcxm@shutcm.edu.cn](mailto:wchcxm@shutcm.edu.cn)


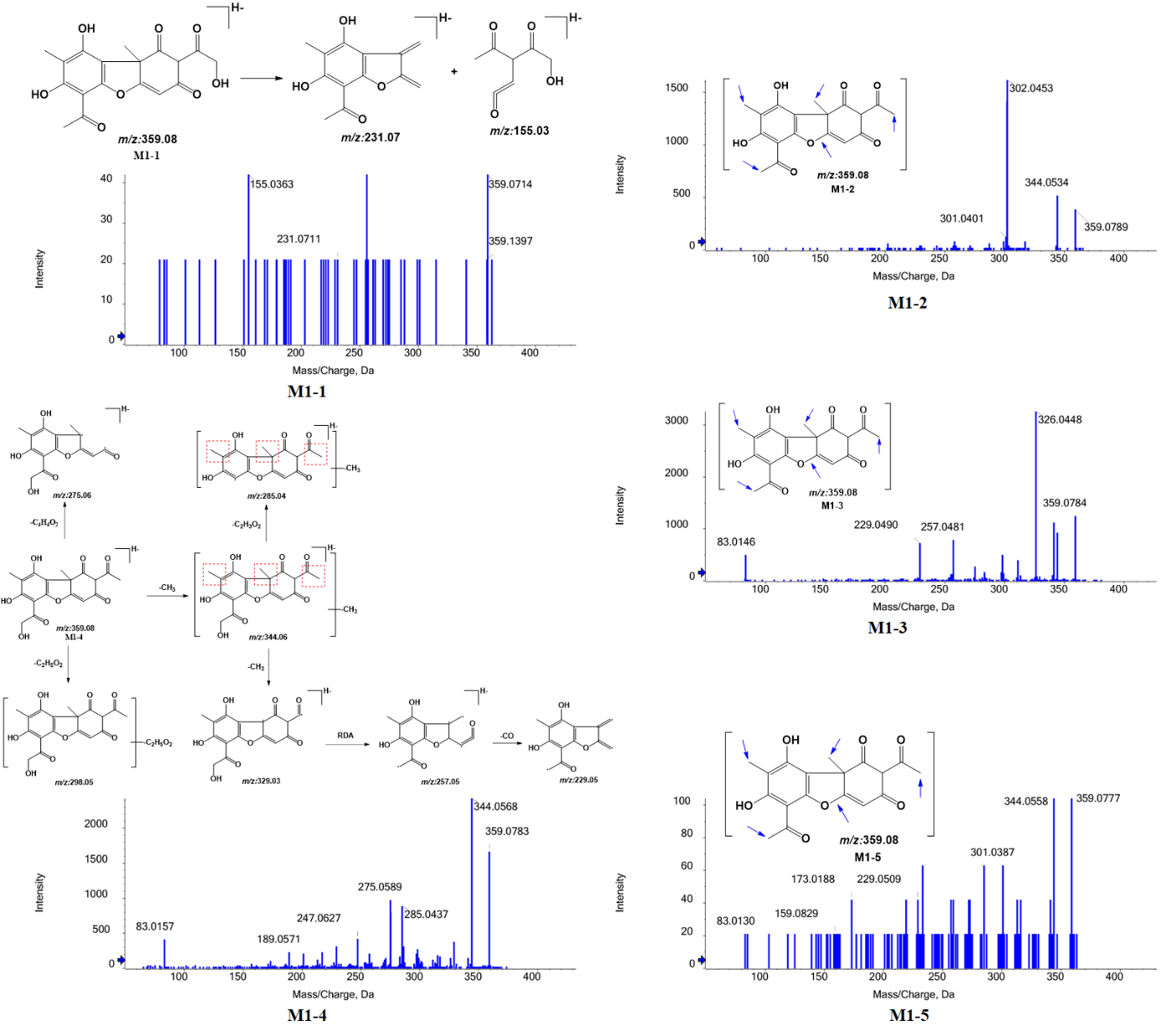


**Supplementary materials Figure S1.** MS/MS spectrum of M1-1 to M1-5 and its proposed fragmentation (part).


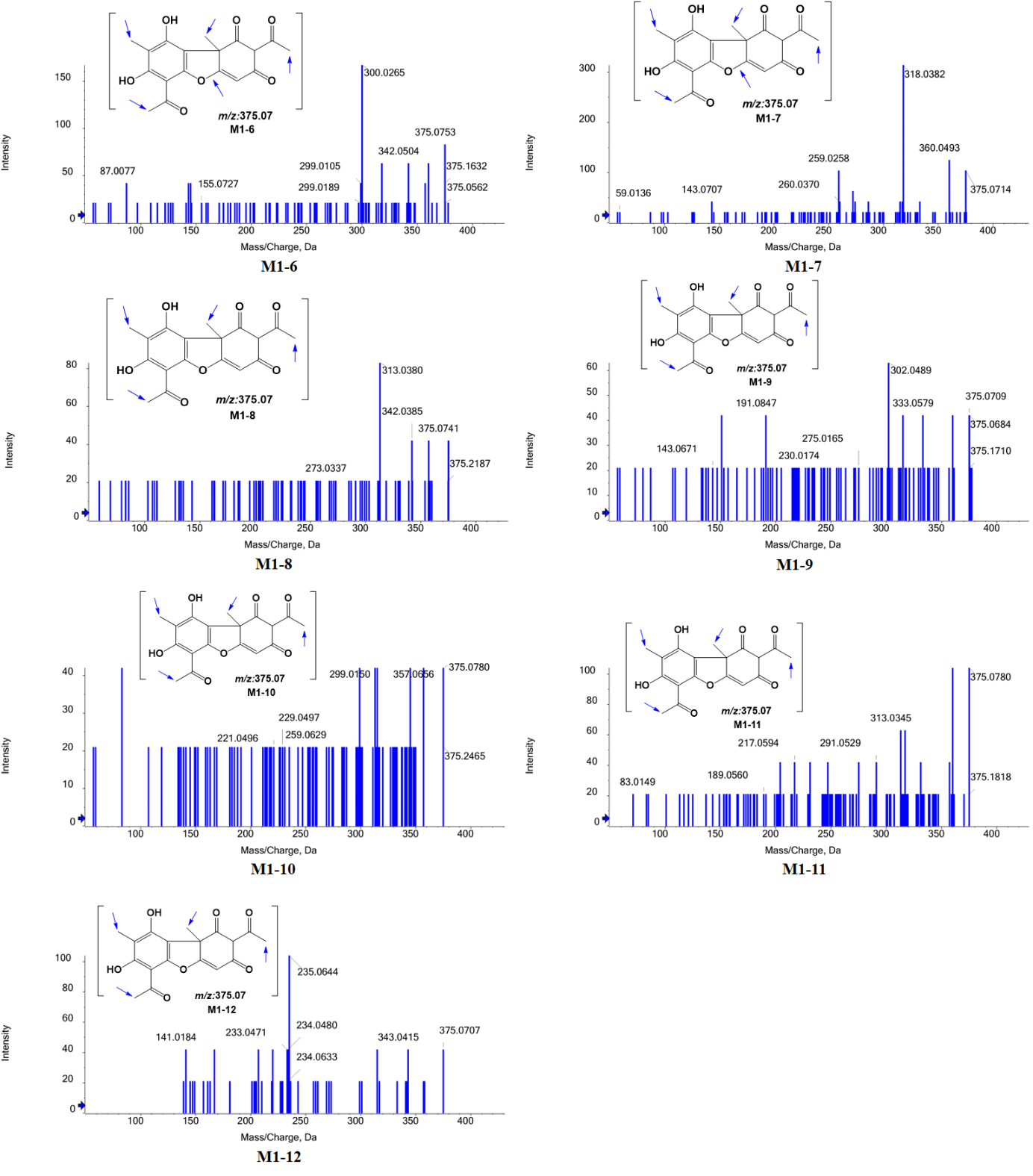


**Supplementary materials Figure S2.** MS/MS spectrum of M1-6 to M1-12.


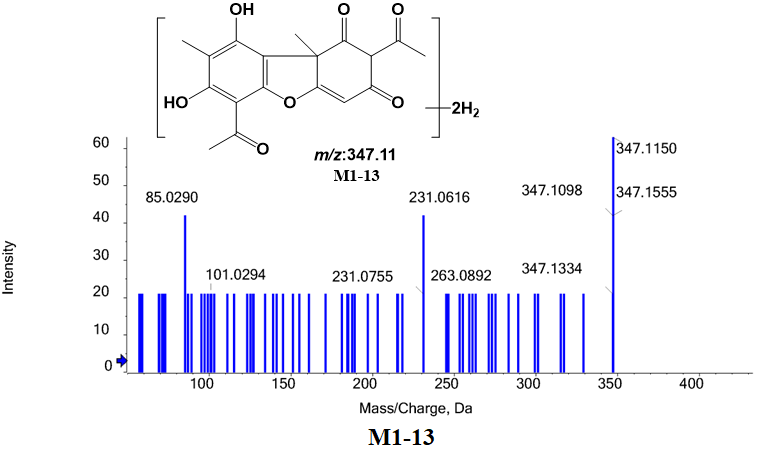


**Supplementary materials Figure S3.** MS/MS spectrum of M1-13.


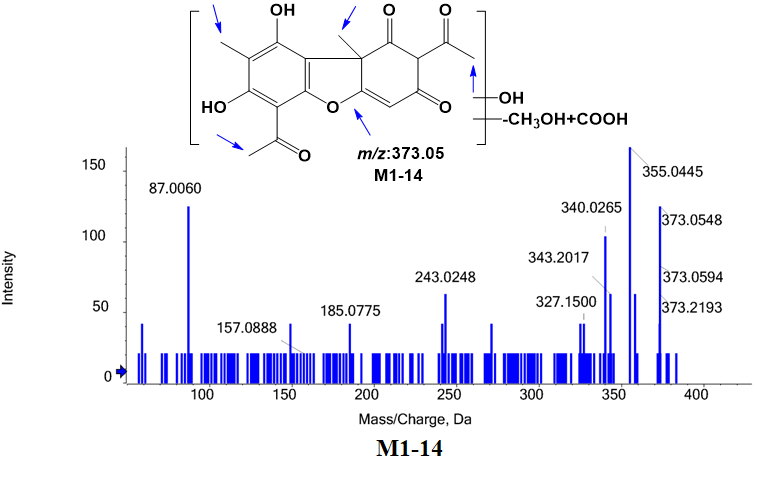


**Supplementary materials Figure S4.** MS/MS spectrum of M1-14.


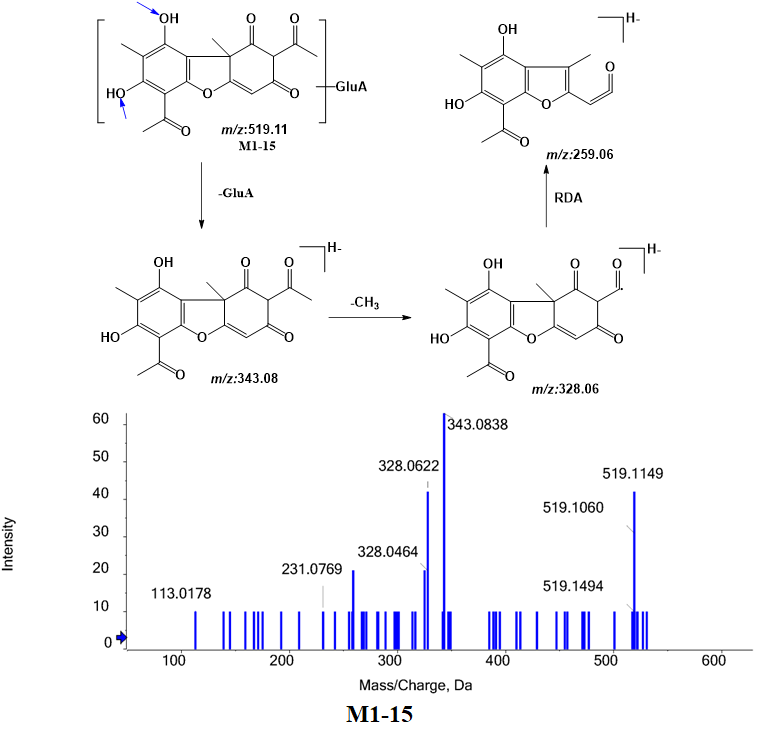


**Supplementary materials Figure S5.** MS/MS spectrum of M1-15 and its proposed fragmentation.


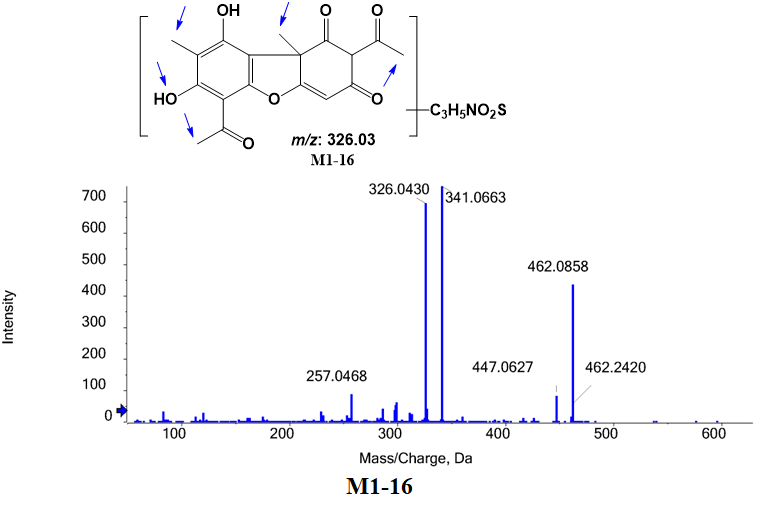


**Supplementary materials Figure S6.** MS/MS spectrum of M1-16.


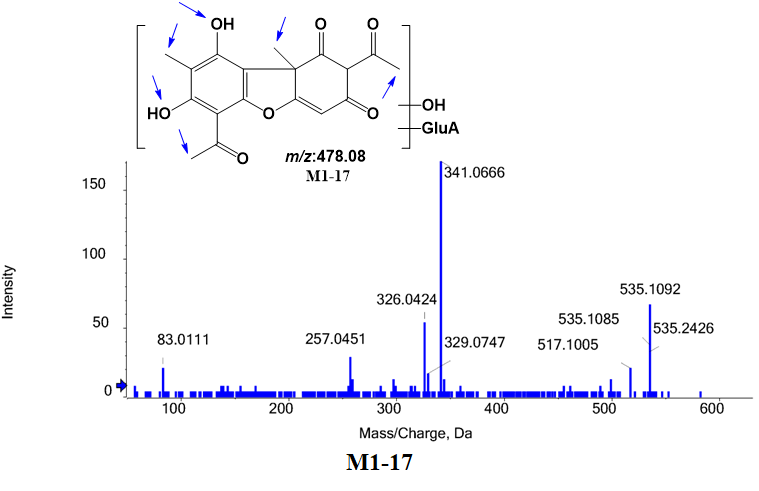


**Supplementary materials Figure S7.** MS/MS spectrum of M1-17.


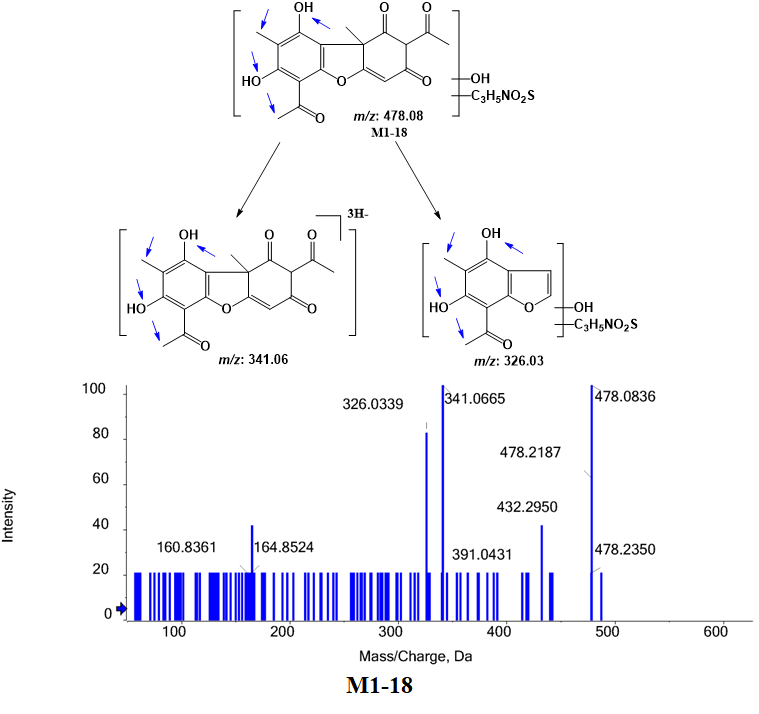


**Supplementary materials Figure S8.** MS/MS spectrum of M1-18 and its proposed fragmentation.


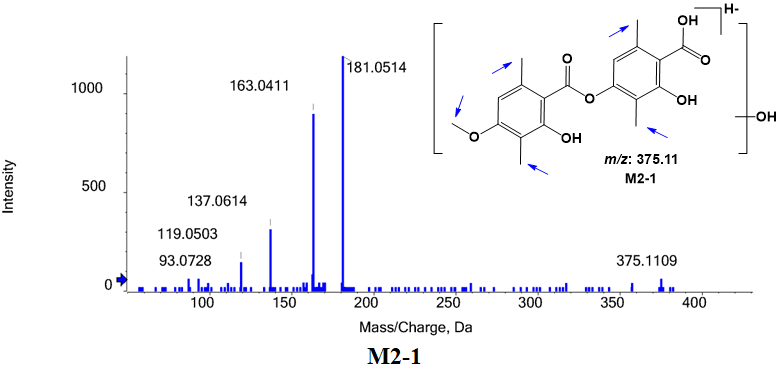


**Supplementary materials Figure S9.** MS/MS spectrum of M2-1.


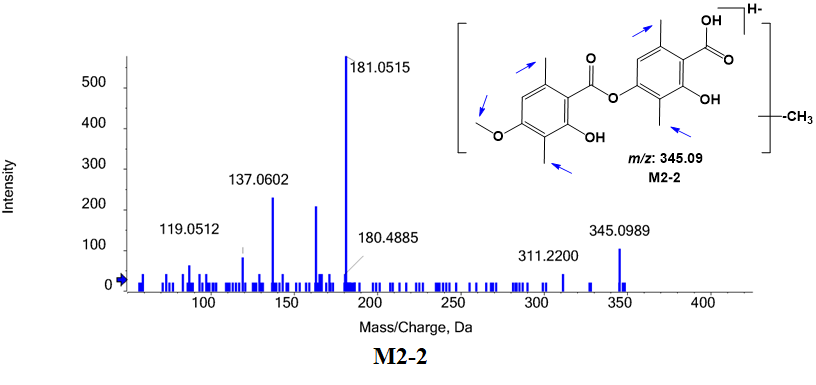


**Supplementary materials Figure S10.** MS/MS spectrum of M2-2.


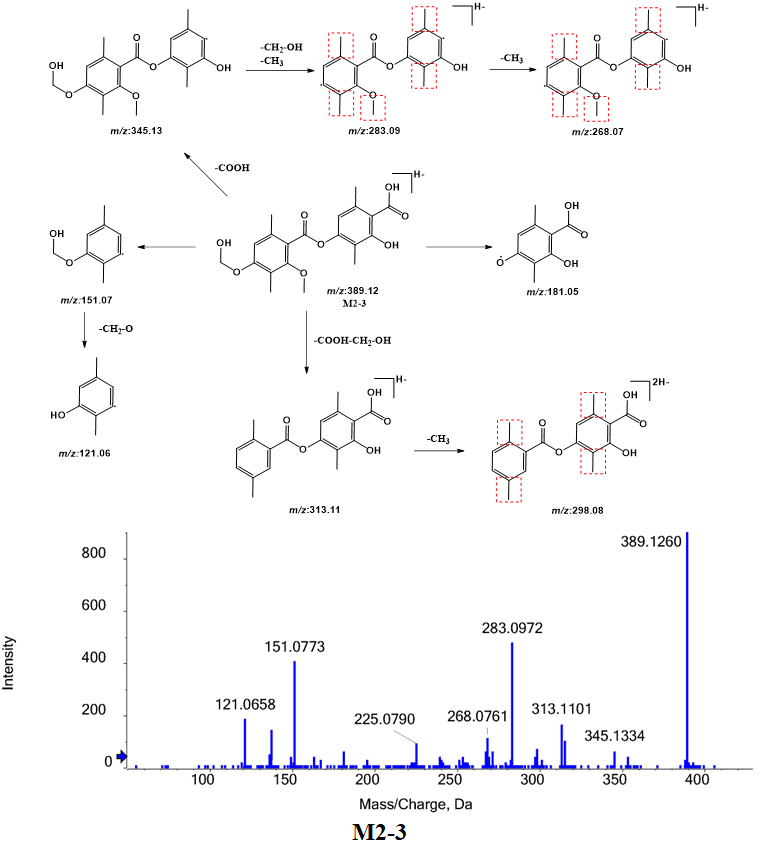


**Supplementary materials Figure S11.** MS/MS spectrum of M2-3 and its proposed fragmentation.


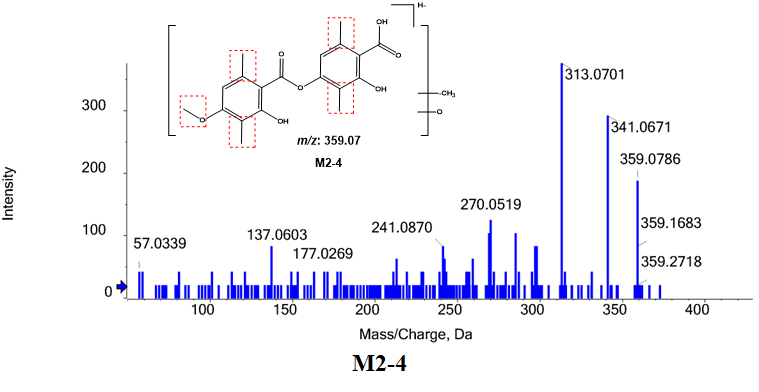


**Supplementary materials Figure S12.** MS/MS spectrum of M2-4.


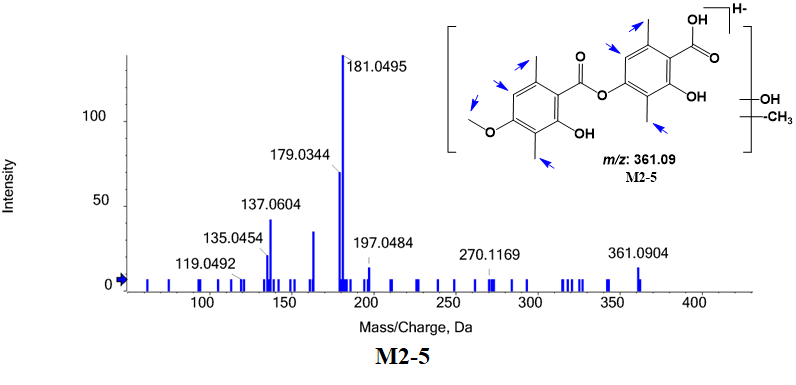


**Supplementary materials Figure S13.** MS/MS spectrum of M2-5.


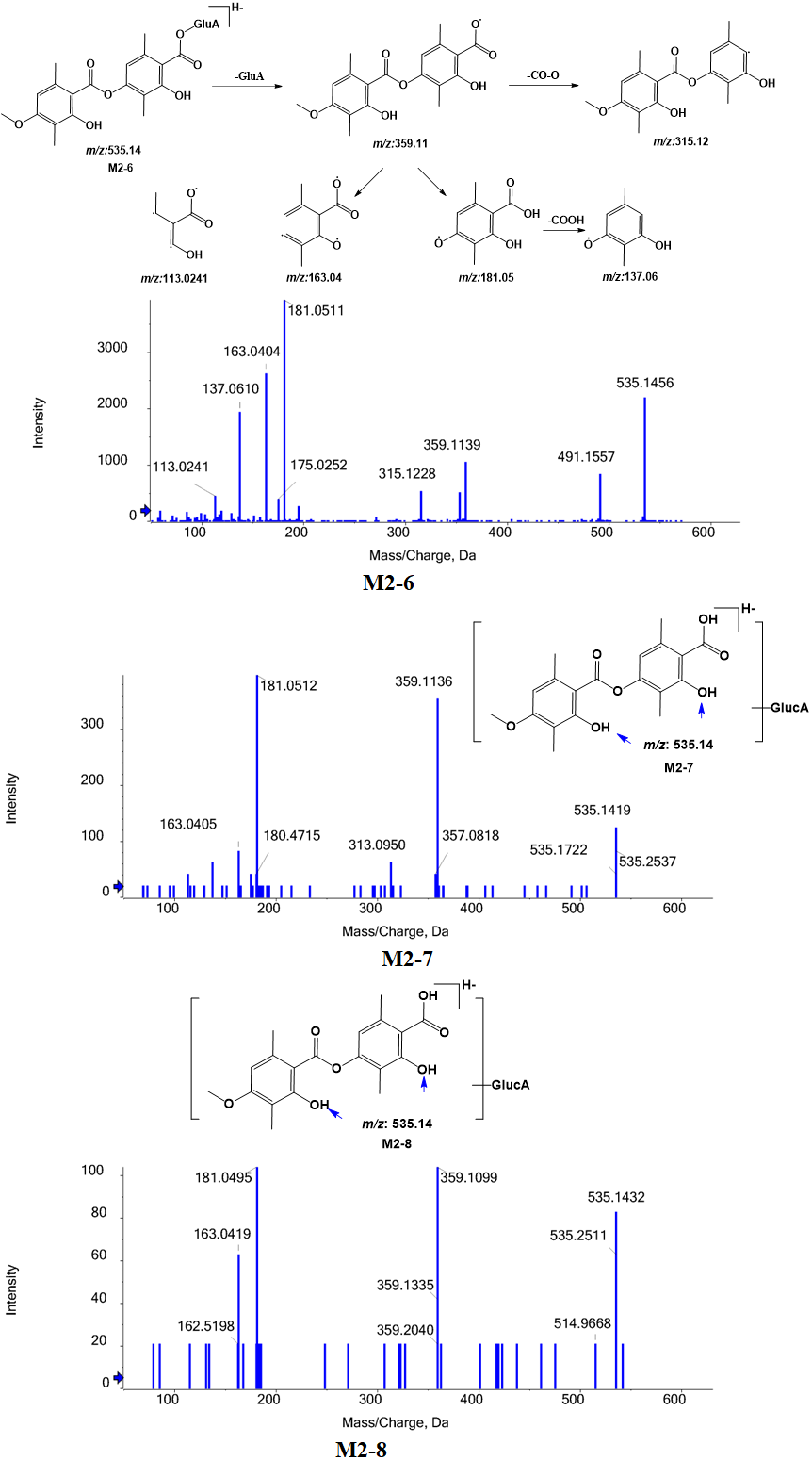


**Supplementary materials Figure S14.** MS/MS spectrum of M2-6 to M2-8 and its proposed fragmentation (part).


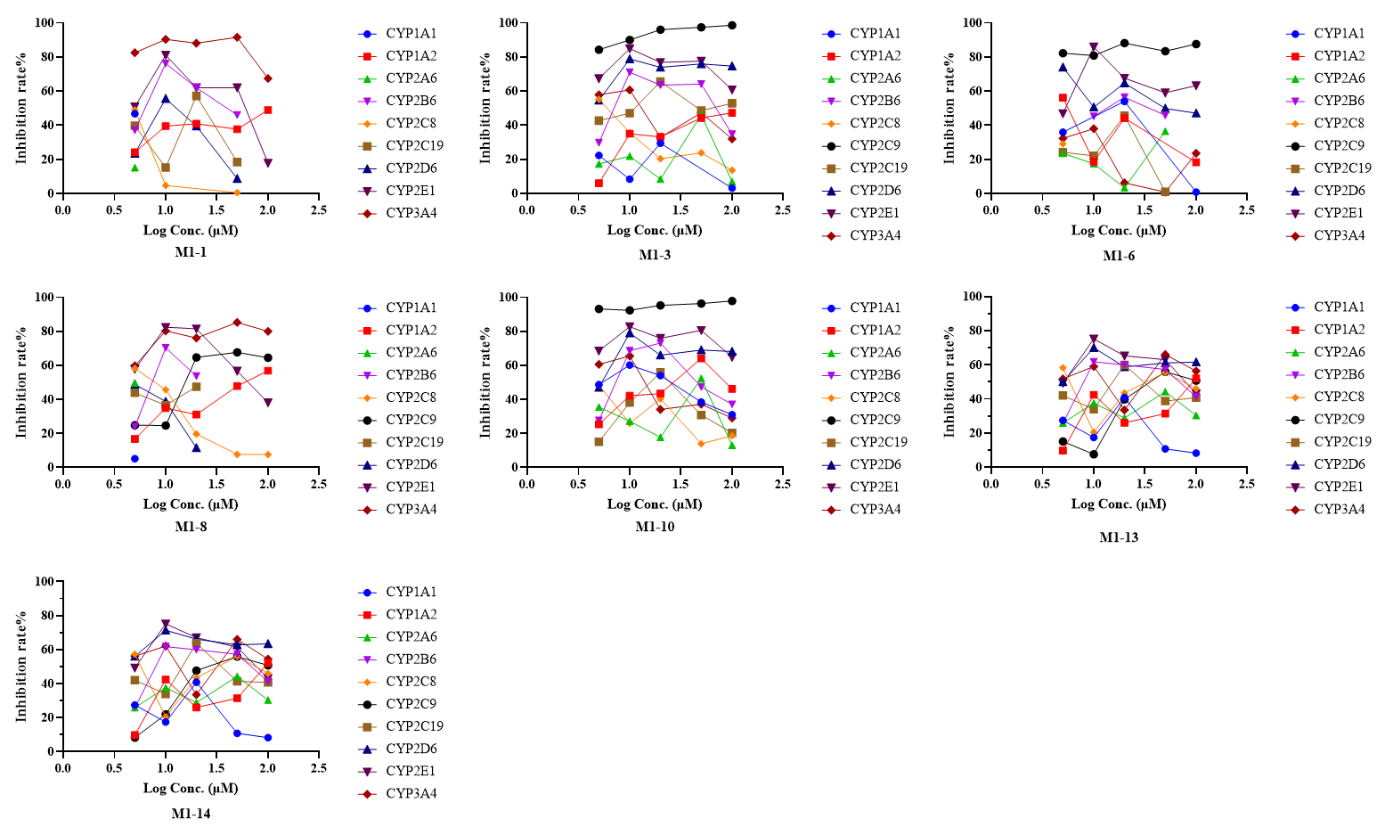


**Supplementary materials Figure S15.** Inhibition rate of UA metabolites in HLM in chemical inhibitors treatment.


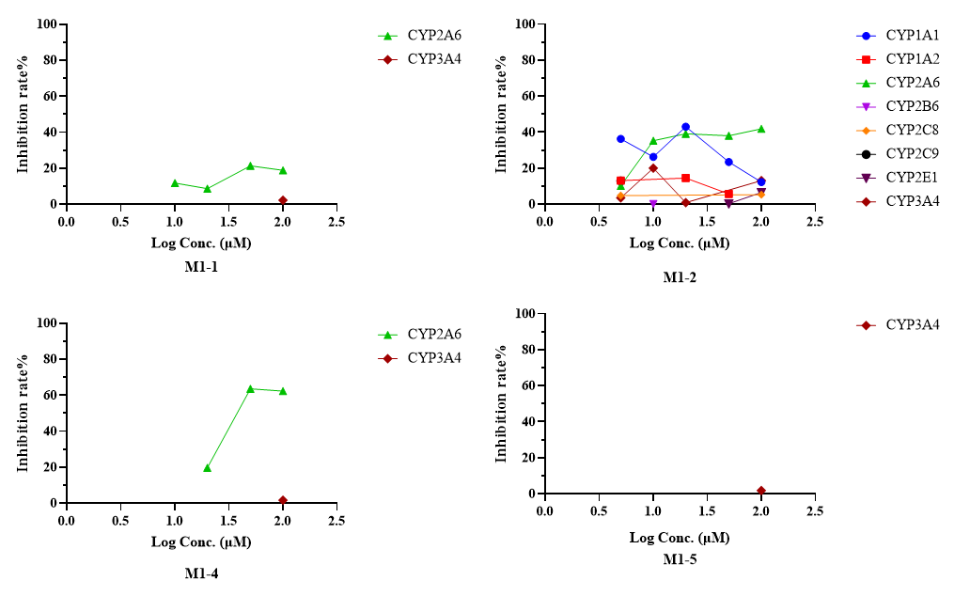


**Supplementary materials Figure S16.** Inhibition rate of BA metabolites in HLM in chemical inhibitors treatment.

**Supplementary materials Table S1.** Toxicity assessment of tamoxifen and mix inhibitors on 3T3 cells (% of NC) (Mean ± SD)

|  | Cell viability in UA sys. | Cell viability in BA sys. |
| --- | --- | --- |
| PC 1 | 5.92 ± 1.17 | 13.50 ± 0.76 |
| PC 2 | 94.6 ± 1.59 | 76.40 ± 1.15 |
|  | Mix Inhibitors + HLMs | Mix Inhibitors + HLMs + NADPH + UDPGA |
| MI | 87.80 ±2.76 | 89.90 ± 1.77 |
